# Autologous stem cell transplantation in major T‐cell lymphoma entities: An analysis by the EBMT Lymphoma Working Party

**DOI:** 10.1002/hem3.70313

**Published:** 2026-02-24

**Authors:** Evgenii Shumilov, Maud Ngoya, Philipp Berning, Irma Khvedelidze, Yasmina Serroukh, Marielle Wondergem, Kate Cwynarski, Lorenz Thurner, Björn E. Wahlin, Robert Zeiser, Carin Hazenberg, Emma Nicholson, Peter Remenyi, Tanja Netelenbos, Stig Lenhoff, Olivier Tournilhac, Keith Wilson, Aloysius Ho, Georg Lenz, Gerald Wulf, Bertram Glass, Peter Dreger, Anna Sureda, Ghandi Damaj, Ali Bazarbachi, Norbert Schmitz

**Affiliations:** ^1^ Department of Medicine A, Hematology and Oncology University Hospital of Muenster Muenster Germany; ^2^ European Society for Blood and Marrow Transplantation, Paris Study Unit, Hôpital Saint‐Antoine Paris France; ^3^ Department of Hematology, Erasmus Medical Center Cancer Institute University Medical Center Rotterdam Rotterdam the Netherlands; ^4^ Department of Hematology Amsterdam University Medical Centers, Cancer Center Amsterdam Amsterdam the Netherlands; ^5^ University College London Hospital's NHS Foundation Trust London UK; ^6^ Department of Internal Medicine I Saarland University Medical School Homburg/Saar Germany; ^7^ Lymphoma section, Hematology Unit Karolinska Universitetssjukhuset Stockholm Sweden; ^8^ Department of Medicine at Huddinge Karolinska Institutet Stockholm Sweden; ^9^ Department of Hematology, Oncology and Stem Cell Transplantation, Faculty of Medicine Freiburg University Medical Center Freiburg Germany; ^10^ Department of Hematology University Medical Center Groningen, University of Groningen Groningen the Netherlands; ^11^ Royal Marsden Hospital London United Kingdom; ^12^ Department of Hematology Central Hospital of Southern Pest, National Institute of Hematology and Infectious Diseases Budapest Hungary; ^13^ Haga Teaching Hospital, Department of Hematology the Hague the Netherlands; ^14^ Department of Hematology Skåne University Hospital Lund Sweden; ^15^ Department of Hematology and Cell Therapy Clermont‐Ferrand University Hospital, University Clermont Auvergne Clermont‐Ferrand France; ^16^ Blood and Bone Marrow Transplantation Programme Cardiff and Vale University Health Board Cardiff UK; ^17^ Department of Hematology Singapore General Hospital and National Cancer Centre Singapore Singapore Singapore; ^18^ Department of Hematology and Medical Oncology University Hospital of Goettingen Goettingen Germany; ^19^ Department of Hematology and Cell Therapy Helios Clinic Berlin‐Buch Germany; ^20^ Department Medicine V University of Heidelberg Heidelberg Germany; ^21^ Clinical Hematology Department, Institut Català d'Oncologia ‐ L'Hospitalet, IDIBELL Universitat de Barcelona Barcelona Spain; ^22^ Centre Hospitalier‐Universitaire, Institut d'Hématologie, Université de Caen Normandie, CNRS, Normandie Université Caen France; ^23^ Bone Marrow Transplantation Program, Department of Internal Medicine American University of Beirut Beirut Lebanon

## Abstract

Autologous stem cell transplantation (auto‐SCT) is an established treatment for peripheral T‐cell lymphoma (PTCL) to consolidate first remission and for patients with relapsed/refractory (r/r) disease. We aimed to examine the outcomes of patients with PTCL NOS, AITL, and ALK‐negative ALCL undergoing auto‐SCT between 2010 and 2022 and reported to EBMT. Adult patients with major T‐cell entities who had received auto‐SCT either up‐front or for r/r disease were included. 2082 patients underwent up‐front and 1249 salvage auto‐SCT. Three‐year progression‐free (PFS) and overall survival (OS) after up‐front auto‐SCT were 55.2% and 73.1%. For r/r patients, 3‐year PFS and OS were 42.6% and 59.5%. In multivariate analysis, male patients, histology other than ALK‐negative ALCL, non‐CR, and higher age at auto‐SCT showed significantly lower PFS and OS after both, up‐front and salvage auto‐SCT, mostly reflecting the higher relapse incidence for these patients. Major outcomes did not significantly change when the analyses were restricted to the patients with PET‐based response at auto‐SCT (*n* = 2062). Auto‐SCT demonstrated excellent outcomes when used up‐front and surprisingly good results in salvage settings. Patients with ALK‐negative ALCL survived significantly better than patients with PTCL NOS or AITL. Male gender, higher age, and non‐CR at auto‐SCT were associated with poor outcomes. Overall, auto‐SCT is a valid treatment option in T‐cell lymphoma where targeted therapies still play a limited role.

## INTRODUCTION

T‐cell lymphomas represent a heterogeneous group of malignancies accounting for about 10% of all lymphomas in the Western world.^1^ The most frequent entities are angioimmunoblastic T‐cell lymphoma (AITL), peripheral T‐cell lymphoma, not otherwise specified (PTCL NOS), anaplastic large cell lymphoma (ALCL), anaplastic lymphoma kinase (ALK)‐negative and ALK‐positive, comprising up to 75% of all peripheral T‐cell lymphoma (PTCL).^2^, ^3^ While patients with ALK‐positive ALCL carry a significantly better prognosis,^4^ the other major entities mostly show an aggressive clinical course with 5‐year overall survival (OS) rates between 30%–50%.^5^, ^6^, ^7^

High‐dose chemotherapy followed by autologous stem cell transplantation (auto‐SCT) is a well‐established procedure, readily available worldwide.^8^, ^9^ Current guidelines recommend auto‐SCT in younger patients responding to first‐line chemotherapy^6^, ^10^ and in relapsed patients who achieved a complete remission after conventional chemotherapy.^11^, ^12^ In both settings, prospective randomized studies comparing auto‐SCT to conventional therapies have not been reported, although three trials are presently being conducted (NCT04489264, NCT06724237, NCT05444712).

We performed a detailed analysis in large numbers of patients evaluating recent outcomes of auto‐SCT for major T‐cell lymphoma entities up‐front and in relapsed/refractory (r/r) disease as reported to the European Society for Blood and Marrow Transplantation (EBMT).

## METHODS

### Study design and data collection

This is a retrospective registry‐based, multi‐center study. Data were provided by the Lymphoma Working Party (LWP) of the EBMT. EBMT is a voluntary group of transplant centers requiring to report all consecutive SCT and follow‐up once a year. All participating institutions are required to obtain written informed consent from patients prior to registration with EBMT, following the Helsinki Declaration. We included adult patients (≥18 years) diagnosed with PTCL NOS, AITL, and ALK‐negative ALCL who had received auto‐SCT either up‐front or in r/r disease as first SCT between January 2010 and December 2022. We retained the diagnosis of AITL, as the re‐naming of the entity was introduced in 2022 only. Baseline and transplantation characteristics as well as outcomes of eligible patients were retrieved from the EBMT registry.

### Definitions

Diagnosis was based on local pathology reports. Patients were staged according to the Ann‐Arbor system. Disease status at transplantation was assessed by local investigators according to standard criteria [(CR, PR, SD (stable disease) and PD (progressive disease)] and classified as chemosensitive (CR/PR), or chemorefractory disease (SD/PD).^13^ While computed tomography (CT) was the standard imaging procedure in earlier years, positron emission tomography (PET) took over more recently. PET was considered for the subgroup of patients with available data.

### Statistics

Endpoints analyzed were PFS (survival without lymphoma relapse or progression), OS (time from transplantation to death from any cause), non‐relapse mortality (NRM) (death without previous relapse), and relapse incidence (RI) (disease recurrence). All outcomes were measured from the day of transplantation. Surviving patients were censored at the time of last contact. Probabilities of OS and PFS were calculated using the Kaplan–Meier method. We calculated cumulative incidences for RI and NRM using a competing risk model, where death was treated as a competing event. Demographics were compared between groups using the chi‐squared test or Fisher's exact test for categorical variables and the Mann–Whitney *U* test for continuous variables. Univariate analyses were performed using the log‐rank test for PFS and OS, while Gray's test was used for competing risk outcome data. Multivariate analyses were performed using the Cox proportional‐hazards regression model. Results are reported as hazard ratios (HR) with a 95% confidence interval (95% CI). All statistical tests were two‐sided with a type I error fixed at 0.05 for factors associated with time‐to‐event outcomes. All analyses were performed using R version 4.3.3 with the R packages survival version 3.5‐8, cmprsk version 2.2‐11 and Hmisc version 5.1‐2. (R Core Team. R: a language for statistical computing. 2014; R Foundation for Statistical Computing).

## RESULTS

### Up‐front auto‐SCT

#### Patient characteristics

2082 patients meeting the eligibility criteria and receiving up‐front auto‐SCT were registered with the EBMT between 2010 and 2022. Major baseline characteristics are presented in Table 1. The patient population included AITL (*n* = 820; 39.4%), PTCL‐NOS (*n* = 765; 36.7%), and ALK‐negative ALCL (*n* = 497; 23.9%). Two‐thirds of the patients were men, the median age was 56 years, and 78.4% had a Karnofsky index ≥90% at auto‐SCT. International Prognostic Index (IPI) (*n* = 1080 patients) scores at diagnosis were as follows: IPI 1 22.8%; IPI 2 26.2%; IPI 3 31.8%; IPI 4–5 19.3%.

**Table 1:** Major characteristics of patients undergoing up‐front and depending on the histology.

Variable in up‐front auto‐SCT	Up‐front auto‐SCT	AITL	PTCL NOS	ALK‐neg. ALCL	*P*‐value
	***n*** = **2082 (100%)**	***n*** = **820 (39.4%)**	***n*** = **765 (36.7%)**	***n*** = **497 (23.9%)**	
Median age at auto‐SCT (range) [IQR], years	56.46 (18.3–77.3) [48.2–63.2]	58.9 (21.4–77.3) [51.2–64.2]	55.5 (19.4–73.6) [47.6–62.6]	53.5 (18.3–75.8) [44.7–61.2]	**<0.0001**
Median time from first diagnosis to auto‐SCT (range) [IQR], months	6.23 (0.5–12) [5.2–7.6]	6.1 (2.6–12) [5.1–7.3]	6.3 (0.7–11.9) [5.2–7.9]	6.3 (0.5–11.8) [5.2–7.6]	0.057
Sex, *n* (%)					
Female	778 (37.4)	342 (41.8)	274 (35.8)	162 (32.7)	0.0022
Male	1302 (62.6)	477 (58.2)	491 (64.2)	334 (67.3)	
Missing	2	1	0	1	
Ann Arbor stage, *n* (%)					
I–II	55 (16.3)	9 (7.6)	26 (17.2)	20 (29.4)	**0.0023**
III	120 (35.5)	50 (42)	53 (35.1)	17 (25)	
IV	163 (48.2)	60 (50.4)	72 (47.7)	31 (45.6)	
Missing	1744	701	614	429	
Induction treatment prior up‐front auto‐SCT, *n* (%)					
Antracycline‐based therapy	1784 (87.5)	693 (89.8)	628 (88.1)	404 (84.9)	**<0.0001**
Other	298 (12.5)	79 (10.2)	85 (11.9)	72 (15.1)	
Missing	0	48	52	21	
Antracycline‐based therapy in combination with brentuximab vedotin, *n* (%)	111/497 (22.3)	26 (3.2)	26 (3.4)	111 (22.3)	**<0.0001**
Remission status at auto‐SCT, *n* (%)					
CR1	1572 (75.5)	623 (76)	569 (74.4)	380 (76.5)	0.6482
PR1	510 (24.5)	197 (24)	196 (25.6)	117 (23.5)	
PET remission status at auto‐SCT, *n* (%)	1324 (63.6%)				
Negative	1055 (79.7)	416 (79.5)	369 (78.5)	270 (81.6)	0.5671
Positive	269 (20.3)	107 (20.5)	101 (21.5)	61 (18.4)	
Use of CT at auto‐SCT confirmed, *n* (%)	1572 (75.5%)	627 (76.5)	551 (72)	394 (79.3)	**0.0099**
Use of CT at auto‐SCT unknown, *n* (%)	510 (24.5%)	193 (23.5)	214 (28)	103 (20.7)	
IPI index score at diagnosis, *n* (%)					
Low risk	246 (22.8)	70 (16.4)	81 (22.5)	95 (32.5)	**<0.0001**
Low‐intermediate risk	283 (26.2)	108 (25.2)	91 (25.3)	84 (28.8)	
High‐intermediate risk	343 (31.8)	158 (36.9)	112 (31.1)	73 (25)	
High risk	208 (19.3)	92 (21.5)	76 (21.1)	40 (13.7)	
Missing	1002	392	405	205	
Karnofsky index at auto‐SCT, *n* (%)					
<90	418 (21.6)	173 (23)	149 (20.8)	96 (20.6)	0.4851
≥90	1515 (78.4)	578 (77)	568 (79.2)	369 (79.4)	
Missing	149	69	48	32	
Preparatory regimen, *n* (%)					
BEAM	1521 (73.6)	613 (75.6)	547 (71.8)	361 (73.2)	
BEAM‐like	194 (9.4)	63 (7.7)	90 (11.8)	41 (8.3)	
BuCyFlu/BuCy	6 (0.3)	4 (0.5)	2 (0.3)	0 (0)	
Other	345 (16.7)	131 (16.2)	123 (16.1)	91 (18.5)	
Missing	16	9	3	4	

		Total up‐front SCT	[2010–2015]	[2016–2022]	
Year of transplantation for all patients		2082	285	1797	*P*‐value
Time of SCT (yrs)	Median (range) [IQR]	2018 (2010–2022) [2016–2020]	2013 (2010–2015) [2011–2015]	2019 (2016–2022) [2017–2021]	<0.0001
Time of SCT (yrs)	[2016, 2022]	1797 (86.3)	0 (0)	1797 (100)	<0.0001
	[2010, 2015]	285 (13.7)	285 (100)	0 (0)	

Abbreviations: AITL, angioimmunoblastic T‐cell lymphoma; ALK‐negative ALCL, anaplastic lymphoma kinase‐negative anaplastic large cell lymphoma; Auto‐SCT, autologous stem cell transplantation; BEAM, carmustine, etoposide, cytarabine, melphalan; BuCy, busulfan, cyclophosphamide; BuCyFlu, busulfan, cyclophosphamide, fludarabine; CR, complete remission; CT, computed tomography; IPI, international prognostic index; PET, positron emission tomography; PR, partial remission; PTCL NOS, peripheral T‐cell lymphoma not otherwise specified; SD/PD, stable disease/progressive disease.

1784 patients (87.5%) had undergone anthracycline‐based chemotherapy prior to auto‐SCT. 111 of 497 ALK‐negative ALCL patients (22.3%) had received brentuximab vedotin (BV) with induction therapy. 1572 (75.5%) and 510 (24.5%) patients underwent up‐front auto‐SCT in CR and PR, respectively. Metabolic remission status at auto‐SCT was available in 1324 patients (63.6%) with 1055 of them (79.7%) being in complete metabolic remission (CMR). BEAM or BEAM‐like preparatory regimens were utilised most frequently (83.0%). The remission status at auto‐SCT in patients with PTCL NOS (CR1 for 74.4%), AITL (CR1 76%), and ALK‐negative ALCL (CR1 76.5%) did not differ significantly (*P* = 0.65).

The numbers of up‐front auto‐SCT per year increased more than sixfold over time: from 37 cases in 2010 to 238 in year 2016 (Supporting Information S1: Table S1). The percentage of patients autografted in CR increased from 70.8% in 2010–2012 to 78.1% in 2019–2022. Median age at SCT, from 54.9 in 2010–2012 to 57.1 in 2019–2022, as well as the proportion of patients aged ≥65, from 4.7% in 2010–2012 to 53% in 2019–2022, increased over time as well (Supporting Information S1: Table S1).

#### Outcomes and prognostic factors in patients undergoing up‐front auto‐SCT

**Figure 1:**
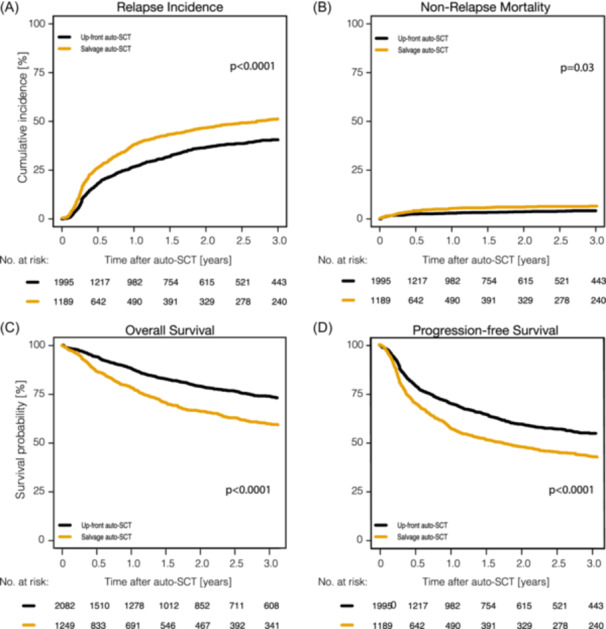
**Outcomes of all patients of the study undergoing up‐front and salvage auto‐SCT**. **(A)** Relapse incidence; **(B)** Non‐relapse mortality; **(C)** Overall survival; **(D)** Progression‐free survival. Auto‐SCT, autologous stem cell transplantation.

Major outcomes of patients receiving up‐front auto‐SCT are shown in Figure 1 and Supporting Information S1: Table S2. The median follow‐up post‐auto‐SCT was 2.1 years (range, 2.0–2.3 years). The 1‐ and 3‐year PFS rates were 70.2% (95% CI, 68%–72.4%) and 55.2% (95% CI, 52.5%–57.9%). The corresponding OS rates were 87.4% (95% CI, 85.7%–88.9%) and 73.1% (95% CI, 70.5%–75.4%). RI was 26.8% (95% CI: 24.7%–29%) and 40.6% (95% CI: 37.9%–43.2%) at 1‐ and 3‐years post‐auto‐SCT; NRM was 3.0% (95% CI: 2.3%–3.8%) and 4.2% (95% CI: 3.2%–5.3%) at 1 and 3 years, respectively. 303 patients (14.5%) received allo‐SCT after failing up‐front auto‐SCT. The estimated 5‐year PFS, OS, RI, and NRM following up‐front auto‐SCT was 45.5%, 65.2%, 48.6% and 5.9%, accordingly (Supporting Information S1: Figure S1).

#### Univariate analyses in patients with up‐front auto‐SCT

**Figure 2:**
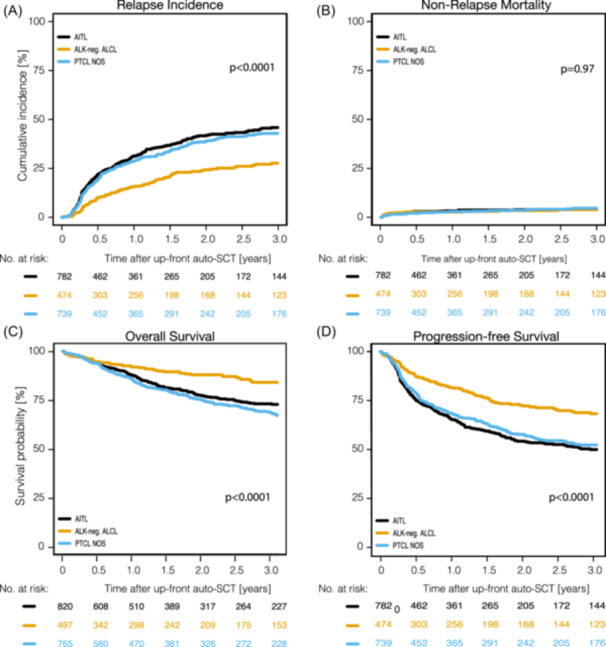
**Outcomes of up‐front auto‐SCT depending on histology**. **(A)** Relapse incidence; **(B)** Non‐relapse mortality; **(C)** Overall survival; **(D)** Progression‐free survival. ALK‐negative ALCL, anaplastic lymphoma kinase‐negative anaplastic large cell lymphoma; auto‐SCT, autologous stem cell transplantation; AITL, angioimmunoblastic T‐cell lymphoma; PTCL NOS, peripheral T‐cell lymphoma not otherwise specified.

OS, PFS, RI, and NRM across subgroups are shown in Figure 2, Supporting Information S1: Figure S2–9 and Table 2. Patients in first CR survived significantly better than those undergoing auto‐SCT in first PR due to a significantly lower 3‐year RI: 35.9% (95% CI: 32.9%–38.9%) vs. 54.5% (95% CI: 48.9%–59.7%) (*P* < 0.0001) (Supporting Information S1: Figure S2 and Table 2) This observation held true when patients with CMR were compared to patients not in CMR at auto‐SCT (Supporting Information S1: Figure S3). No significant differences in survival were documented between CR and CMR, as well as PR and non‐CMR patients (Supporting Information S1: Figure S4‐5).

**Table 2:** Univariate analysis of factors influencing OS, PFS, RI, or NRM after up‐front auto‐SCT.

		OS			PFS			RI			NRM		
			*P*‐value	HR (95% CI)		*P*‐value	HR (95% CI)		*P*‐value	HR (95% CI)		*P*‐value	HR (95% CI)
Remission status at SCT	CR	76.6% [73.7–79.1]	**<0.0001**	1	59.7% [56.5–62.7]	**<0.0001**	1	35.9% [32.9–38.9]	**<0.0001**	1	4.4% [3.3–5.7]	0.466	1
	PR	62.9% [57.4–67.9]		1.71 (1.41–2.07)	42.1% [36.7–47.4]		1.72 (1.48–2.01)	54.5% [48.9–59.7]		1.84 (1.56–2.16)	3.5% [1.9–5.8]		0.96 (0.56–1.63)
Year of SCT	[2010–2015]	73% [66.7–78.3]	0.792	1	57% [50.3–63.2]	0.741	1	39.2% [32.8–45.5]	0.793	1	3.8% [1.8–6.8]	0.978	1
	[2016–2022]	73.3% [70.5–75.9]		1.03 (0.81–1.32)	54.9% [51.9–57.8]		1.03 (0.85–1.25)	40.9% [38–43.8]		1.04 (0.84–1.27)	4.2% [3.2–5.4]		1.01 (0.56–1.81)
Lymphoma	AITL	72.7% [68.6–76.4]	**<0.0001**	1	50.3% [45.8–54.5]	**<0.0001**	1	45.9% [41.5–50.1]	**<0.0001**	1	3.9% [2.6–5.6]	0.973	1
	ALK‐neg. ALCL	84.1% [79.3–87.8]		0.59 (0.44–0.79)	68.5% [62.8–73.5]		0.54 (0.44–0.67)	27.7% [22.6–32.9]		0.51 (0.41–0.64)	3.9% [2.2–6.3]		0.86 (0.48–1.53)
	PTCL NOS	67.2% [62.8–71.2]		1.18 (0.97–1.44)	52.6% [48.1–56.8]		0.9 (0.77‐1.05)	42.9% [38.6–47.1]		0.9 (0.76–1.06)	4.6% [3–6.6]		0.92 (0.56–1.52)
Karnofsky Index	<90	70.1% [63.7–75.6]	**0.0341**	1	54.7% [47.9–61]	0.864	1	39.5% [33–45.9]	0.582	1	5.9% [3.5–9]	0.128	1
	≥90	73.6% [70.7–76.3]		0.78 (0.62–0.98)	55.7% [52.5–58.7]		0.98 (0.82–1.19)	40.4% [37.3–43.4]		1.04 (0.85–1.27)	3.9% [2.9–5.2]		0.68 (0.41–1.13)
Sex	Male	71.1% [67.8–74.2]	**0.001 46**	1	52.5% [49–55.9]	**0.005 57**	1	43.3% [39.9–46.7]	**0.009 45**	1	4.2% [3–5.6]	0.574	1
	Female	76.3% [72.3–79.8]		0.72 (0.59–0.88)	59.7% [55.3–63.8]		0.81 (0.69–0.94)	36.3% [32.1–40.4]		0.8 (0.68–0.94)	4% [2.6–5.9]		0.83 (0.52–1.32)
Age at SCT	18–56	81.5% [78.2–84.3]	**<0.0001**	1	60.4% [56.4–64.1]	**<0.0001**	1	37.3% [33.6–41.1]	**0.0167**	1	2.3% [1.4–3.6]	**<0.0001**	1
	>56	65.5% [61.7–69]		1.92 (1.58–2.33)	50.5% [46.6–54.2]		1.37 (1.19–1.59)	43.6% [39.8–47.3]		1.26 (1.08–1.46)	6% [4.4–7.8]		3.08 (1.86–5.12)
IPI at diagnosis	(0–2 points)	79.9% [75–84]	**<0.0001**	1	64.1% [58.6–69]	**<0.0001**	1	31.6% [26.6–36.6]	**<0.0001**	1	4.4% [2.5–6.9]	0.938	1
	(3–5 points)	67.6% [62.3–72.3]		1.82 (1.38–2.42)	48.2% [42.7–53.5]		1.61 (1.3–1.98)	47.8% [42.3–53]		1.69 (1.35–2.11)	4% [2.4–6.2]		1.06 (0.56–2.01)

Abbreviations: AITL, angioimmunoblastic T‐cell lymphoma; ALK‐neg, ALCL, anaplastic lymphoma kinase‐negative anaplastic large cell lymphoma; auto‐SCT, autologous stem cell transplantation; CR, complete remission; IPI, international prognostic index; NRM, non‐relapse mortality; OS, overall survival; PFS, progression‐free survival; PR, partial remission; PTCL NOS, peripheral T‐cell lymphoma not otherwise specified; RI, relapse incidence.

#### Different outcomes in major entities

While the 3‐year NRM rate did not differ significantly across all three entities (4.2% range, 3.9%–4.6%) (*P* = 0.97), RI was significantly lower in ALK‐negative ALCL ([3‐year RI: 27.7% (95% CI: 22.6%–32.9%)) in comparison to PTCL NOS [(3‐year RI: 42.9% (95% CI: 38.6–47.1%)] and AITL [(3‐year RI: 45.9% (95% CI: 41.5–50.1%)] (*P* < 0.0001). Entity‐specific analyses showed the highest PFS‐ and OS‐rates occurring in patients with ALK‐negative ALCL [(3‐year PFS 68.5% (95% CI, 62.8%–73.5%); OS 84.1% (95% CI, 79.3%–87.8%)]. PFS rates at 3 years were 52.6% (95% CI, 48.1%–56.8%), and 50.3% (95% CI, 45.8%–54.5%), OS rates were 67.2% (95% CI, 62.8%–71.2%) and 72.7% (95% CI, 68.6%–76.4%) in PTCL NOS and AITL, respectively (*P* < 0.0001 for both, PFS and OS) (Table 2 and Figure 2). Patients with ALK‐negative ALCL showed better survival and lower RI compared to patients with the other entities when undergoing auto‐SCT in CR/CMR and PR at the time of auto‐SCT (Supporting Information S1: Figure S6–8). Finally, the estimated 5‐year PFS, OS, RI, and NRM in ALK‐negative ALCL, AITL, and PTCL NOS after up‐front auto‐SCT was 58.5% vs. 41.3% vs. 42.4% (*P* < 0.0001), 75.8% vs. 66% vs. 58.6% (*P* < 0.0001), 34.7% vs. 53.7% vs. 51.4% (*P* < 0.0001), and 6.8% vs. 4.9% vs. 6.2% (*P* = 0.97) (Supporting Information S1: Figure S9), respectively.

#### Prognostic factors in patients undergoing up‐front auto‐SCT

**Figure 3:**
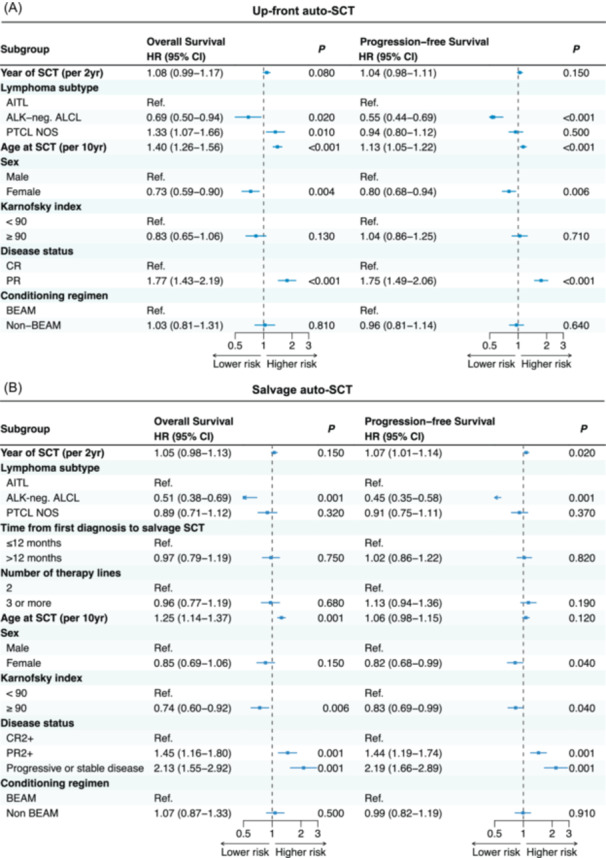
**Prognostic factors influencing the outcomes of up‐front (A) and salvage (B) auto‐SCT in a multivariate Cox‐regression model**. Auto‐SCT, autologous stem cell transplantation; AITL, angioimmunoblastic T‐cell lymphoma; ALK‐negative ALCL, anaplastic lymphoma kinase‐negative anaplastic large cell lymphoma; BEAM, carmustine, etoposide, cytarabine, melphalan; CR, complete remission; PR, partial remission; PTCL NOS, peripheral T‐cell lymphoma not otherwise specified; SD/PD, stable disease/progressive disease.

To investigate factors influencing the outcomes of auto‐SCT, we applied a multivariate model including the following variables: year of SCT, sex, histology, age at SCT, remission status at auto‐SCT, Karnofsky index, and type of conditioning (Figure 3A and Supporting Information S1: Table S3).

#### Progression‐free survival

The multivariate analysis showed that female sex was associated with a better PFS (HR 0.8, 95% CI 0.68–0.94, *P* = 0.006). Increased age (HR 1.13, 95% CI 1.05–1.22, *P* < 0.001) and PR at upfront auto‐SCT (HR 1.75, 95% CI 1.49–2.06, *P* < 0.001) were associated with lower PFS. ALK‐negative ALCL (HR 0.55, 95% CI 0.44–0.69, *P* < 0.001) was associated with a better PFS.

#### Overall survival

Multivariate analysis showed that female sex was associated with a better OS (HR 0.73, 95% CI 0.59–0.9, *P* = 0.003). Increased age (HR 1.40, 95% CI 1.26–1.56, *P* < 0.001) and PR at upfront auto‐SCT (HR 1.77, 95% CI 1.43–2.19, *P* < 0.001) were associated with lower OS. Patients with PTCL NOS (HR 1.33, 95% CI 1.07–1.66, *P* = 0.010) showed a lower OS when compared to patients with ALK‐negative ALCL (HR 0.69, 95% CI 0.50–0.94, *P* = 0.020) when using AITL as reference.

#### Relapse incidence

Multivariate analysis showed that female sex (HR 0.79, 95% CI 0.66–0.93, *P* = 0.006) and ALK‐negative ALCL (HR 0.5, 95% CI 0.39–0.64, *P* < 0.001) were associated with a lower relapse incidence. PR at auto‐SCT (HR 1.89, 95% CI 1.59–2.24, *P* < 0.001) was associated with significantly increased relapse rate.

#### Non‐relapse mortality

For NRM, only age at auto‐SCT (HR 2.08, 95% CI 1.57–2.75, *P* < 0.001) was associated with increased risk.

### Salvage auto‐SCT

#### Patient characteristics

Major characteristics are summarized in Table 3. Overall, 1249 patients underwent salvage auto‐SCT. Median age was 58 years at transplantation with 65.6% of patients being male. Sixty‐nine percent of the patients had received two and the remaining patients (30.6%) three or more lines of therapy prior to auto‐SCT. Remission status at auto‐SCT was CR in 58.7%, PR in 31.7%, and SD/PD in 9.7%. At auto‐SCT, patients with ALK‐negative ALCL and AITL presented more frequently in CR2+ than patients with PTCL NOS: 61.9% for both versus 54.1% (*P* = 0.021). 738 out of 1249 patients (59.1%) had PET imaging results available at auto‐SCT and 461 of them were in CMR (62.5%).

**Table 3:** Major characteristics of patients undergoing salvage auto‐SCT and depending on the histology.

	Salvage auto‐SCT	AITL	PTCL NOS	ALK‐neg. ALCL	
Variable in salvage auto‐SCT	*n* = 1249 (100%)	*n* = 402 (32.2%)	*n* = 519 (41.6%)	*n* = 328 (26.3%)	*P*‐value
Median age at auto‐SCT (range) [IQR], years	58.0 (18.7–85.7) [49.1–64.4]	61.3 (24.2–82.3) [53.6–65.9]	57.5 (18.7–85.7) [48.1–63.9]	54.2 (19.2–74.7) [44.1–62.4]	**<0.0001**
Median time from first diagnosis to auto‐SCT (range) [IQR], months	10.74 (2–301.1) [7.1–17.5]	10 (3.5–254) [6.7–16.7]	10.7 (2–301.1) [7.3–16.8]	11.4 (2.9–160.6) [7.4–19]	0.0548
Sex, *n* (%)					
Female	429 (34.4)	155 (38.8)	172 (33.2)	102 (31.1)	0.0719
Male	817 (65.6)	245 (61.3)	346 (66.8)	226 (68.9)	
Missing	3	2	1	0	
Ann Arbor stage, *n* (%)					
I–II	60 (20.1)	7 (7.4)	31 (22.3)	22 (33.8)	**0.0009**
III	87 (29.2)	35 (37.2)	39 (28.1)	13 (20)	
IV	151 (50.7)	52 (55.3)	69 (49.6)	30 (46.2)	
Missing	951	308	380	263	
Primary refractory/early relapse vs. late relapse, *n* (%)					
≤12 months	718 (57.5)	245 (60.9)	300 (57.8)	173 (52.7)	0.0818
>12 months	531 (42.5)	157 (39.1)	219 (42.2)	155 (47.3)	
Number of therapy lines prior auto‐SCT, *n* (%)					
Two (2 L)	867 (69.4)	282 (70.1)	362 (69.7)	223 (68)	0.8009
Three and more (3 L+)	382 (30.6)	120 (29.9)	157 (30.3)	105 (32)	
Remission status at auto‐SCT, *n* (%)					
CR2/PR2	790 (63.3)	263 (65.4)	323 (62.2)	204 (62.2)	0.2828
CR3+/PR3+	338 (27.1)	111 (27.6)	139 (26.8)	88 (26.8)	
Progressive or stable disease		28 (7)	57 (11)	36 (11)	
CR2	537 (43.0)	179 (44.5)	212 (40.8)	146 (44.5)	0.0867
CR3+	196 (15.7)	70 (17.4)	69 (13.3)	57 (17.4)	
PR2	253 (20.3)	84 (20.9)	111 (21.4)	58 (17.7)	
PR3+	142 (11.4)	41 (10.2)	70 (13.5)	31 (9.5)	
Progressive or stable disease	121 (9.7)	28 (7)	57 (11)	36 (11)	
PET remission status at auto‐SCT, *n* (%)	738 (59.1%)				
Negative	461 (62.5%)	161 (68.8)	166 (55.7)	134 (65)	**0.0055**
Positive	277 (37.5%)	73 (31.2)	132 (44.3)	72 (35)	
Use of CT at auto‐SCT confirmed, *n* (%)	814 (65.2%)	270 (67.2)	327 (63)	217 (66.2)	0.3836
Use of CT at auto‐SCT unknown, *n* (%)	435 (34.8%)	132 (32.8)	192 (37)	111 (33.8)	
IPI index score at diagnosis, *n* (%)					
Low risk	106 (19.1)	18 (10.9)	41 (19.2)	47 (26.7)	**0.0003**
Low‐intermediate risk	164 (29.5)	41 (24.8)	73 (34.1)	50 (28.4)	
High‐intermediate risk	161 (29.0)	66 (40)	50 (23.4)	45 (25.6)	
High risk	124 (22.3)	40 (24.2)	50 (23.4)	34 (19.3)	
Missing	694	237	305	152	
Karnofsky index at auto‐SCT, *n* (%)					
<90	396 (30.1)	110 (29.3)	151 (30.9)	92 (29.7)	0.8729
≥90	968 (69.9)	265 (70.7)	338 (69.1)	218 (70.3)	
Missing	75	27	30	18	
Preparatory regimen, *n* (%)					
BEAM	820 (66.3)	280 (70.2)	326 (63.3)	214 (66.5)	
BEAM‐like	154 (12.4)	40 (10)	76 (14.8)	38 (11.8)	
BuCyFlu/BuCy	9 (0.7)	3 (0.8)	4 (0.8)	2 (0.6)	
Other	253 (20.5)	76 (19)	109 (21.2)	68 (21.1)	
Missing	13	3	4	6	

		Total salvage SCT	[2016–2022]	[2010–2015]	
Year of transplantation for all patients		1249	943	306	P‐value
Time of SCT (yrs)	Median (range) [IQR]	2017 (2010–2022) [2016–2020]	2019 (2016–2022) [2017–2020]	2013 (2010–2015) [2011–2014]	<0.0001
Time of SCT (yrs)	[2016, 2022]	943 (75.5)	943 (100)	0 (0)	<0.0001
	[2010, 2015]	306 (24.5)	0 (0)	306 (100)	

Abbreviations: AITL, angioimmunoblastic T‐cell lymphoma; ALK‐negative ALCL, anaplastic lymphoma kinase‐negative anaplastic large cell lymphoma; auto‐SCT, autologous stem cell transplantation; BEAM, carmustine, etoposide, cytarabine, melphalan; BuCy, busulfan, cyclophosphamide; BuCyFlu, busulfan, cyclophosphamide, fludarabine; CR, complete remission; CT, computed tomography; IPI, international prognostic index; PET, positron emission tomography; PR, partial remission; PTCL NOS, peripheral T‐cell lymphoma not otherwise specified; SD/PD, stable disease/progressive disease.

Prior to salvage auto‐SCT, 78.7% of patients underwent BEAM high‐dose therapy or similar. The numbers of auto‐SCT per annum increased over time as did the median age of patients (54 years in 2010–2012 and 59 years in 2019–2022) and the proportion of patients aged ≥65 (Supporting Information S1: Table S1). 193 of 1249 patients (15.5%) received allo‐SCT after failing auto‐SCT.

#### Outcomes and prognostic factors in patients undergoing auto‐SCT for relapsed/refractory disease

Major outcomes of patients receiving auto‐SCT for r/r disease are shown in Table 4, Supporting Information S1: Table S4, and Figure 1. The median follow‐up was of 2.6 years (95% CI: 2.2–3.0). With a median follow‐up of 2.6 years (95% CI: 2.2–3.0), 1‐year and 3‐year PFS rates were 57.1% (95% CI: 53.9%–60.1%) and 42.6% (95% CI: 39.2%–46.0%). The corresponding OS rates were 77.9% (95% CI, 75.2%–80.3%) and 59.5% (95% CI, 56.1%–62.8%), respectively. RI was 37.9% (95% CI: 34.9%–41.0%) and 51.2% (95% CI: 47.7%–54.5%) at 1 and 3 years post‐auto‐SCT. NRM was 5.0% (95% CI: 3.8%–6.5%) and 6.2% (95% CI: 4.8%–7.9%) at 1 and 3 years. The estimated 5‐year PFS, OS, RI, and NRM after salvage auto‐SCT were 36.5%, 51.8%, 55.8%, and 7.7% (Supporting Information S1: Figure S1), respectively.

**Table 4:** Univariate analysis of factors influencing OS, PFS, RI, or NRM after salvage auto‐SCT.

		OS			PFS			RI			NRM		
			*P*‐value	HR (95% CI)		*P*‐value	HR (95% CI)		*P*‐value	HR (95% CI)		*P*‐value	HR (95% CI)
Conditioning	BEAM	61.3% [57–65.3]	0.0603	1	42.4% [38.2–46.5]	0.571	1	52.7% [48.4–56.9]	0.43	1	4.9% [3.4–6.8]	**0.009 26**	1
	Non‐ BEAM	55.6% [49.5–61.2]		1.2 (0.99–1.48)	43.2% [37.4–48.9]		1.05 (0.88–1.24)	48% [42.1–53.6]		0.97 (0.80–1.16)	8.8% [6–12.3]		1.8 (1.15–2.92)
Time diagnosis to SCT	≤12 months	61.4% [56.9–65.5]	0.872	1	45.3% [40.9–49.6]	0.812	1	49.4% [45–53.7]	0.905	1	5.2% [3.6–7.3]	0.405	1
	>12 months	56.9% [51.4–62.1]		1.02 (0.84–1.23)	38.8% [33.5–44]		1.02 (0.87–1.20)	53.6% [48.2–58.8]		0.99 (0.83–1.19)	7.6% [5.2–10.6]		1.2 (0.76–1.92)
Disease status at SCT	CR2+	65.5% [61–69.7]	**<0.0001**	1	50.3% [45.8–54.7]	**<0.0001**	1	44.7% [40.3–49.1]	**<0.0001**	1	5% [3.4–7]	**<0.0001**	1
	PR2+	54.2% [48.1–59.9]		1.43 (1.16–1.77)	35.6% [29.9–41.3]		1.43 (1.20–1.71)	59.5% [53.4–65.1]		1.50 (1.24–1.80)	4.9% [2.8–7.8]		0.94 (0.53–1.68)
	SD/PD	43% [32.1–53.4]		2.03 (1.52–2.72)	21.4% [13.2–31]		2.08 (1.62–2.67)	60.8% [49.4–70.4]		1.82 (1.37–2.42)	17.8% [10.8–26.3]		3.85 (2.19–6.77)
Year of SCT	[2010–2015]	60.3% [54.2–65.9]	0.764	1	43.7% [37.6–49.6]	0.339	1	49.7% [43.5–55.6]	0.376	1	6.6% [4.1–10]	0.901	1
	[2016–2022]	59.4% [55.1–63.4]		1.03 (0.84–1.28)	42.4% [38.3–46.4]		1.09 (0.91–1.31)	51.6% [47.4–55.6]		1.10 (0.9–1.33)	6% [4.4–7.9]		1.06 (0.63–1.78)
Lymphoma	AITL	54.3% [47.9–60.3]	**<0.0001**	1	35.5% [29.6–41.3]	**<0.0001**	1	57.2% [50.9–63]	**<0.0001**	1	7.4% [4.8–10.7]	0.338	1
	ALK‐ neg. ALCL	73.8% [67.4–79.1]		0.48 (0.36–0.64)	62.4% [55.6–68.4]		0.49 (0.39–0.62)	31.3% [25.2–37.4]		0.47 (0.36–0.60)	6.3% [3.7–9.9]		0.64 (0.35–1.16)
	PTCL NOS	54.9% [49.5–59.9]		0.93 (0.75–1.15)	36.4% [31.5–41.4]		0.97 (0.81–1.16)	58.3% [53–63.2]		1.01 (0.84–1.23)	5.3% [3.4–7.8]		0.67 (0.39–1.15)
Karnofsky Index	<90	51.4% [44.8–57.6]	**<0.0001**	1	37.1% [31–43.2]	**0.0106**	1	54.8% [48.3–60.8]	0.179	1	8.1% [5.3–11.8]	**0.0246**	1
	≥90	62.5% [58.3–66.5]		0.65 (0.53–0.80)	44.9% [40.6–49]		0.79 (0.66–0.95)	49.5% [45.3–53.6]		0.84 (0.69–1.02)	5.6% [4–7.7]		0.54 (0.33–0.87)
Sex	Male	56.9% [52.5–61]	0.0992	1	39.5% [35.3–43.6]	**0.0197**	1	54.7% [50.5–58.8]	**0.006 11**	1	5.8% [4.1–7.8]	0.344	1
	Female	64.3% [58.6–69.5]		0.84 (0.68–1.03)	48.4% [42.5–54]		0.81 (0.68–0.97)	44.7% [38.9–50.4]		0.77 (0.64–0.93)	6.9% [4.5–10]		1.16 (0.72–1.87)
Age at SCT	18–56	67.4% [62.4–72]	**<0.0001**	1	47.4% [42.2–52.3]	**0.008 83**	1	48.2% [43–53.1]	0.21	1	4.5% [2.8–6.8]	**0.006 58**	1
	>56	53.1% [48.4–57.6]		1.62 (1.33–1.98)	38.8% [34.4–43.3]		1.25 (1.06–1.47)	53.5% [48.9–58]		1.16 (0.98–1.39)	7.6% [5.6–10.1]		2.11 (1.27–3.52)
No. of prior therapy lines	2	60% [55.8–63.9]	0.996	1	44% [39.9–48.1]	**0.0482**	1	50.6% [46.4–54.6]	0.139	1	5.4% [3.9–7.3]	0.476	1
	≥3	58.5% [52.1–64.3]		1 (0.81–1.23)	39.5% [33.7–45.4]		1.19 (1.0–1.41)	52.4% [46.3–58.2]		1.18 (0.98–1.42)	8% [5.3–11.5]		1.28 (0.78–2.07)

Abbreviations: AITL, angioimmunoblastic T‐cell lymphoma; ALK‐neg. ALCL, anaplastic lymphoma kinase‐negative anaplastic large cell lymphoma; auto‐SCT, autologous stem cell transplantation; CR, complete remission; IPI, international prognostic index; NRM, non‐relapse mortality; OS, overall survival; PFS, progression‐free survival; PR, partial remission; PTCL NOS, peripheral T‐cell lymphoma not otherwise specified; RI, relapse incidence; SD/PD, stable disease/progressive disease.

#### Univariate analysis in patients with auto‐SCT for relapsed/refractory disease

As for patients autografted up‐front, outcomes after auto‐SCT for r/r disease were significantly better for patients in CR than in PR or SD/PD. This was mainly due to a significantly lower RI among CR patients: 3‐year RI was 44.7% (95% CI: 40.3–49.1%), 59.5% (95% CI: 53.4%–65.1%), and 60.8% (95% CI: 49.4%–70.4%) in patients transplanted in CR, PR, or <PR (*P* < 0.0001) (Table 4 and Supporting Information S1: Figure S10).

The same trend was found for patients undergoing SCT in CMR (Supporting Information S1: Figure S9) while there were no significant differences in outcomes post‐auto‐SCT for those with CR determined by CT or PET (Supporting Information S1: Figure S12).

#### Different outcomes in major entities

**Figure 4:**
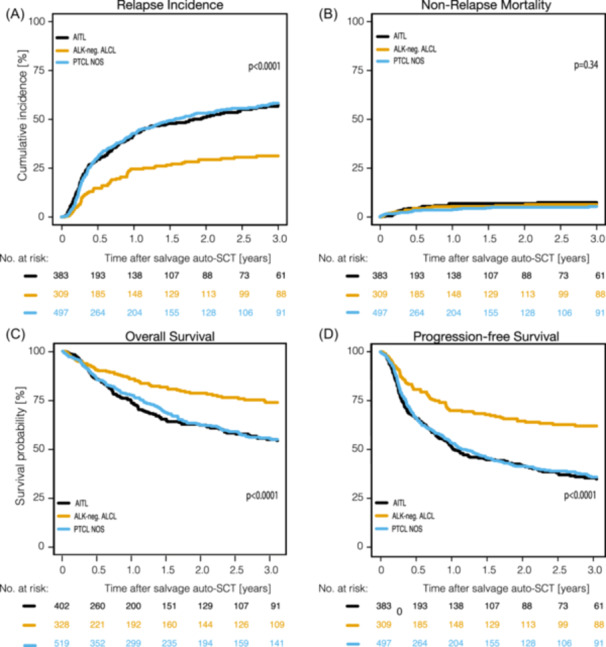
**Outcomes of salvage auto‐SCT depending on histology**. AITL, angioimmunoblastic T‐cell lymphoma; ALK‐negative ALCL, anaplastic lymphoma kinase‐negative anaplastic large cell lymphoma; auto‐SCT, autologous stem cell transplantation; PTCL NOS, peripheral T‐cell lymphoma not otherwise specified.

OS, PFS, RI, NRM in PTCL NOS, AITL, and ALK‐negative ALCL are shown in Figure 4, Supporting Information S1: Figure S10–15, and Table 4. Three‐year NRM did not significantly differ across all major entities (6.2%; range, 5.3%−7.4%) (*P* = 0.338) although being significantly higher in chemorefractory patients (17.8%) (Table 4). In line with our findings in up‐front SCT, 3‐year RI following salvage auto‐SCT was significantly higher in PTCL NOS [(58.3% (CI: 53.0%–63.2%)] and AITL [(57.2% (CI: 50.9%–63.0%)] in comparison to ALK‐negative ALCL [(31.3% (CI: 25.2%–37.4%)] (*P* < 0.0001). Accordingly, 3‐year PFS rates were significantly better in ALK‐negative ALCL [(62.4% (95% CI: 55.6%–68.4%)] than in PTCL NOS [(36.4% (95% CI: 31.5%–41.4%)] and in AITL [(35.5% (95% CI: 29.6%–41.3%)] (*P* < 0.0001). Three‐year OS rates were 73.8% (95% CI, 67.4%–79.1%), 54.9% (95% CI, 49.5%–59.9%), and 54.3% (95% CI, 47.9%–60.3%), for ALK‐negative ALCL, PTCL NOS, and AITL, respectively (*P* < 0.0001) (Table 4 and Figure 4). Additionally, ALK‐negative ALCL patients in CR/CMR and PR showed a significantly better survival and lower RI in comparison to similar patient groups with PTCL NOS and AITL (Supporting Information S1: Figures S13–15). Finally, the estimated 5‐year PFS, OS, RI, and NRM in ALK‐negative ALCL, AITL, and PTCL NOS after salvage auto‐SCT were 57.3% vs. 28.8% vs. 30.2% (*P* < 0.0001), 68.5% vs. 42.1% vs. 48.4% (*P* < 0.0001), 36.3% vs. 60.9% vs. 63.2% (*P* < 0.0001), and 6.3% vs. 10.2% vs. 6.6% (*P *= 0.34) (Supporting Information S1: Figure S16), respectively.

#### Prognostic factors in patients undergoing auto‐SCT for relapsed/refractory disease

To investigate factors influencing the outcomes of auto‐SCT for relapsed/refractory disease, in addition to parameters considered for up‐front patients, we included time to salvage auto‐SCT from diagnosis (≤12 vs. >12 months), number of therapy lines (2 L vs. 3 L+) and remission status (SD/PD vs. PR2+ vs. CR2+) at auto‐SCT in the multivariate model (Supporting Information S1: Table S5 and Figure 3B). Notably, time salvage auto‐SCT from diagnosis and number of therapy lines did not have any impact on the outcomes of salvage auto‐SCT.

#### Progression‐free survival

Female patients (HR 0.82, 95% CI 0.68–0.99, *P* = 0.040) and patients with ALK‐negative ALCL (HR 0.45, 95% CI 0.35–0.58, *P* < 0.001) showed better PFS. Patients in PR (HR 1.44, 95% CI 1.19–1.74, *P* < 0.001) and SD/PD (HR 2.19, 95% CI 1.66–2.89, *P* < 0.001) had lower PFS. Notably, a time period of SCT (per 2 years) influenced PFS as well (HR 1.07, 95% CI 1.01–1.14, *P* = 0.020).

#### Overall survival

PR (HR 1.45, 95% CI 1.16–1.80, *P* < 0.001) and SD/PD patients (HR 2.13, 95% CI 1.55–2.02, *P* < 0.001) showed lower OS, as did patients with increasing age (HR 1.26, 95% CI 1.15–1.38, *P* < 0.001). Patients with ALK‐negative ALCL (HR 0.51, 95% CI 0.38–0.69, *P* < 0.001) and Karnofsky index ≥90% (HR 0.74, 95% CI 0.60–0.92, *P* = 0.006) showed a significantly better OS.

#### Relapse incidence

PR (HR 1.45, 95% CI 1.19–1.77, *P* < 0.001) and SD/PD patients (HR 1.81, 95% CI 1.33–2.45, *P* < 0.001) were associated with higher RI. Female patients (HR 0.78, 95% CI 0.64–0.95, *P* = 0.01) and ALK‐negative ALCL (HR 0.43, 95% CI 0.33–0.57, *P* < 0.001) showed lower RI. A time period of SCT (per 2 years) influenced RI as well (HR 1.09, 95% CI 1.03–1.16, *P* = 0.005).

#### No‐relapse mortality

For NRM, only SD/PD at auto‐SCT (HR 4.38, 95% CI 2.34–8.2, *P* < 0.001) was associated with increased risk.

## DISCUSSION

This study reports major outcomes in a large number of patients with T‐cell lymphoma autografted up‐front or with relapsed/refractory disease in recent years. Notably, we for the first time were able to analyse and compare disease status at transplantation by CT and PET without finding significant differences in outcomes when CT or PET had been used for imaging prior to transplantation. Due to the large number of patients, we were also able to separately analyse outcomes for the major T‐cell lymphoma entities (PTCL NOS, AILT, ALK‐negative ALCL) and identify prognostic factors for key outcomes. Interestingly, results of auto‐SCT differed significantly between entities with patients with ALK‐negative ALCL surviving significantly better than those with other entites, mostly due to a lower relapse incidence after both, up‐front and salvage auto‐SCT. Male gender, higher age, and non‐CR at auto‐SCT were also associated with poor outcomes.

Up‐front auto‐SCT resulted in 3‐year PFS‐ and OS rates of 55.2% and 73.1%, respectively, being similar to PFS‐rates reported in the two largest prospective phase 2 trials, which had shown 3‐year PFS of 48% and 49%, while 3‐year OS in our study was higher than in these phase 2 studies (56% for both).^6^, ^10^, ^14^ This may be explained by the fact that the prospective studies reported intent‐to‐treat data while this analysis reports only patients actually transplanted, missing up to 40% of patients unable to undergo transplantation because of early progression.^6^, ^10^, ^15^, ^16^ Moreover, both prospective studies included patients with rare entities like enteropathy‐associated and hepatosplenic T‐cell lymphoma which generally show poor outcomes after auto‐SCT. Finally, at least one third of our patients failing up‐front auto‐SCT underwent salvage allo‐SCT which has been demonstrated to be associated with improved OS in the recently published long‐term follow‐up of the AATT study. In AATT, 7‐year PFS and ‐OS rates following up‐front auto‐SCT were reported at 50% and 72%, respectively, very well matching the results of our retrospective analysis.^17^ Older age and transplantation not being in remission are known risk factors for auto‐SCT confirmed in this study. The question of whether auto‐SCT should be offered to transplant‐eligible patients reaching CR (and PR) with first‐line therapy is being addressed in the TRANSCRIPT‐study (LYSA, NCT05444712) and 2 other prospective trials (NCT06724237, NCT04489264). Two of these studies include only CR patients while our results suggest that patients in PR and/or those with non‐CMR might also benefit from up‐front auto‐SCT.

The prognosis of T‐cell lymphoma patients failing first‐line therapy remains very poor with a median life expectancy of few months. Recent phase 1/2 studies in r/r T‐cell lymphomas involving new drugs failed to meet their endpoints and mostly gave disappointing results.^18^, ^19^, ^20^ Therefore, it was surprising and encouraging to find that auto‐SCT for chemosensitive patients with r/r disease resulted in 3‐year PFS and OS rates at 42.6% and 59.5%, largely in line with smaller series reported by the Japanese Society for Transplantation and Cellular Therapy (JSTCT; *n* = 448)^21^ and the Center for International Blood and Marrow Transplant Research (CIBMTR; *n* = 115).^22^ Male gender, higher age, and refractory disease at auto‐SCT were associated with inferior OS and PFS in multivariable analysis while early vs. late (≤ vs. >12 months) relapse and the number of prior therapy lines (2 vs. 3+) did not have significant impact. Relapse rates were not significantly different between in patients with PR, chemorefractory disease (SD/PD), and PET‐positive imaging at salvage auto‐SCT, while ALK‐negative ALCL patients again performed significantly better when compared with groups of PTCL NOS and AITL patients with identical remission status. Only patients in CR/CMR have a realistic chance of long‐term survival after auto‐SCT while allo‐SCT should be strongly considered in the remaining cases.^12^, ^17^, ^22^ Planning for salvage auto‐SCT will include the possibility not to achieve a remission with salvage therapy. Such patients should not proceed to auto‐SCT but rather receive allo‐SCT.

In the present study, patients with ALK‐negative ALCL showed a significantly better outcome for both, up‐front and salvage auto‐SCT, as compared with PTCL NOS and AITL. Similar observations were reported from studies involving lower numbers of patients, namely the NLG‐T01 study,^6^ and a retrospective CIBMTR^22^ analysis. Other studies, however, did not confirm these observations. Importantly, we show that the better survival of ALK‐negative ALCL patients are driven by a significantly lower RI, also when comparing CR/CMR and PR patients separately. When comparing ALCL with PTCL NOS and AITL patients, the former were younger and presented less frequently with IPI scores ≥3 at diagnosis. Moreover, one fifth of ALCL patients had received BV‐containing induction therapy showing a significant survival benefit^23^ particularly for patients consolidative up‐front auto‐SCT.^24^

The present study has limitations inherent to any retrospective analysis. Most importantly, we cannot know how many patients initially deemed transplant candidates did not proceed to transplantation because of disease progression or toxicity of induction and salvage therapies. For such analyses, we refer to the recently published final results of the AATT study. Notably, the results of this study and the prospective randomized AATT study done with significantly lower number of patients are remarkably similar when patients actually transplanted are compared. Another limitation of the study is the lack of central pathology review for the cases analyzed and the lack of control group. Furthermore, response assessment was done by local investigators. A centralized review of end‐of‐treatment PET and/or CT could not be done. As the group of patients with ALK‐negative ALCL included a higher proportion of patients with limited‐stage disease, lower IPI scores, and BV‐containing induction therapy, these factors may have influenced the favorable outcomes of these patients. Unique strengths of this study in contrast to previous analyses are the large patient numbers and the fact that the response evaluation was based on CT but also on state‐of‐the‐art PET imaging.

In conclusion, this analysis of a large international cohort of patients diagnosed with AITL, PTCL NOS, and ALK‐negative ALCL demonstrates that auto‐SCT is a surprisingly effective treatment for PTCL patients. Best results can be expected for patients autografted in first CR but also patients transplanted in CR after salvage therapy benefit from the auto‐SCT with limited toxicity. Consistently, patients with ALK‐negative ALCL show better outcomes than patients with PTCL NOS and AITL. In patients with refractory disease transient remissions generally are not durable, and allo‐SCT should strongly be recommended to such patients. The development of targeted agents and immunotherapeutic strategies including chimeric antigen receptor T‐cells deserves further attention and may challenge transplantation in the future.

## AUTHOR CONTRIBUTIONS

**Evgenii Shumilov**: Conceptualization; writing—original draft; methodology; data curation; investigation; validation. **Maud Ngoya**: Conceptualization; writing—original draft; software; methodology; data curation; formal analysis; project administration; resources. **Philipp Berning**: Conceptualization; software; methodology; data curation; writing—review and editing. **Irma Khvedelidze**: Methodology; software; data curation; formal analysis; project administration. **Yasmina Serroukh**: Resources; data curation; writing—review and editing. **Marielle Wondergem**: Resources; data curation; writing—review and editing. **Kate Cwynarski**: Resources; data curation; writing—review and editing. **Lorenz Thurner**: Resources; data curation; writing—review and editing. **Björn Engelbrekt Wahlin**: Resources; data curation; writing—review and editing. **Robert Zeiser**: Resources; data curation; writing—review and editing. **Carin Hazenberg**: Resources; data curation; writing—review and editing. **Emma Nicholson**: Resources; data curation; writing—review and editing. **Peter Remenyi**: Resources; data curation; writing—review and editing. **Tanja Netelenbos**: Resources; data curation; writing—review and editing. **Stig Lenhoff**: Resources; data curation; writing—review and editing. **Olivier Tournilhac**: Resources; data curation; writing—review and editing. **Keith Wilson**: Resources; data curation; writing—review and editing. **Aloysius Ho**: Resources; data curation; writing—review and editing. **Georg Lenz**: Resources; data curation; writing—review and editing; supervision. **Gerald Wulf**: Resources; data curation; writing—review and editing. **Bertram Glass**: Resources; data curation; writing—review and editing. **Peter Dreger**: Resources; data curation; writing—review and editing. **Anna Sureda**: Resources; data curation; writing—review and editing; supervision; project administration. **Ghandi Damaj**: Resources; data curation; writing—review and editing; conceptualization; investigation; supervision; project administration. **Ali Bazarbachi**: Resources; writing—original draft; data curation; writing—review and editing; project administration; supervision; conceptualization; methodology; investigation. **Norbert Schmitz**: Writing—original draft; data curation; resources; writing—review and editing; project administration; supervision; conceptualization; methodology; investigation.

## CONFLICT OF INTEREST STATEMENT

K. Cwynarski: Consulting/Advisory Role: Abbvie, Roche, Autolus, Sobi, KITE, Takeda, BMS, Atara, Gilead, Janssen; Speakers’ Bureau: Roche, Takeda, KITE, Gilead, BMS; Conferences/Travel support: Roche, Takeda, KITE, Janssen, BMS. E. Nicholson: Honoraria: KITE/Gilead, BMS/Celgene, J&J, Sanofi. Travel Support: KITE/Gilead, Amgen, Novartis. Research Support: KITE/Gilead. DSMB: Autolus. K. Wilson: Honoraria: Gilead Kite. Travel support: Gilead Kite. Research support: Gilead Kite (to institution). G. Lenz: received research grants not related to this manuscript from AGIOS, AQUINOX, AstraZeneca, Bayer, Celgene, Gilead, Janssen, MorphoSys, Novartis, F. Hoffmann‐La Roche Ltd, and Verastem. G.L. received honoraria not related to this study from ADC Therapeutics, AbbVie, Amgen, AstraZeneca, Bayer, BeiGene, BMS, Celgene, Constellation, Genase, Genmab, Gilead, Hexal/Sandoz, Immagene, Incyte, Janssen, Karyopharm, Lilly, Miltenyi, MorphoSys, MSD, NanoString, Novartis, PentixaPharm, Pierre Fabre, F. Hoffmann‐La Roche Ltd, and Sobi. P. Dreger: consultancy for AbbVie, AstraZeneca, Beigene, BMS, Gilead, Miltenyi (all to institution); speaker honoraria from AbbVie, AstraZeneca, BeiGene, BMS, Gilead, Riemser, Roche (all to institution); meeting attendance support from Beigene and Gilead; Participation on a Data Safety Monitoring Board for Novartis. P Dreger is current chairman of the German Working Group for Hematopoietic Stem Cell Transplantation and Cellular Therapy (DAG‐HSZT). A. Sureda: Consultancy: Takeda, Abbvie, GenMab, MSD, BMS Celgene, Gilead Kite, Allogene, Autolus, Roche, Novartis; Speakers Bureau: Takeda; Travel: Janssen, Takeda, Gilead Kite, Abbvie. Speaker honoraria: Takeda, BMS Celgene, GenMab, Abbvie, Janssen, Gilead Kite, Vertex, Sanofi; Research support: Takeda, BMS Celgene; A. Sureda is the current President of the EBMT. G. Damaj: Honoraria: Takeda, Amgen. Travel support: Takeda. Research Support: Takeda, Ideogen A. Bazarbachi: Speaker bureau or advisory board: Novartis, Roche, Sanofi, Jazz, Adienne, Astellas, Takeda, Hikma, Celgene, Jansen, MSD, Abbvie, Pfizer and Amgen. Research support: Novartis, Roche, Takeda, Jansen, Astellas, Celgene, Pfizer, and Amgen. N. Schmitz: Honoraria: Gilead Kite, Roche. Travel support: Beigene, Glilead Kite, Roche Research support: Astra Zeneca, Janssen Other authors report no conflict of interest relevant to the contents of this work.

## FUNDING

This research received no funding.

## Supporting information


**Supplemental Figure S1.** Estimated 5‐year outcomes of all patients of the study undergoing up‐front and salvage auto‐SCT. Auto‐SCT, autologous stem cell transplantation.
**Supplemental Figure S2.** Outcomes of up‐front auto‐SCT depending on remission status at SCT (CR vs. PR). Auto‐SCT, autologous stem cell transplantation; CR, complete remission; PR, partial remission.
**Supplemental Figure S3.** Outcomes of up‐front auto‐SCT depending on metabolic remission status at SCT (CMR vs. non‐CMR). Auto‐SCT, autologous stem cell transplantation; CMR, complete metabolic remission.
**Supplemental Figure S4.** Outcomes of up‐front auto‐SCT depending on CR status determined by CT and PET (CR by CT vs. CMR by PET). Auto‐SCT, autologous stem cell transplantation; CR, complete remission; CT, computed tomography; PET, positron emission tomography; CMR, complete metabolic remission.
**Supplemental Figure S5.** Outcomes of up‐front auto‐SCT depending on non‐CR status determined by CT and PET (PR by CT vs. non‐CMR by PET). Auto‐SCT, autologous stem cell transplantation; CR, complete remission; PR, partial remission; CT, computed tomography; CMR, complete metabolic remission; PET, positron emission tomography.
**Supplemental Figure S6.** Outcomes of up‐front auto‐SCT depending on histology among CR patients at SCT. Auto‐SCT, autologous stem cell transplantation; CR, complete remission; ALK‐negative ALCL, anaplastic lymphoma kinase‐negative anaplastic large cell lymphoma; PTCL NOS, peripheral T‐cell lymphoma not otherwise specified; AITL, angioimmunoblastic T‐cell lymphoma.
**Supplemental Figure S7.** Outcomes of up‐front auto‐SCT depending on histology among CMR patients at SCT. Auto‐SCT, autologous stem cell transplantation; CMR, complete metabolic remission; ALK‐negative ALCL, anaplastic lymphoma kinase‐negative anaplastic large cell lymphoma; PTCL NOS, peripheral T‐cell lymphoma not otherwise specified; AITL, angioimmunoblastic T‐cell lymphoma.
**Supplemental Figure S8.** Outcomes of up‐front auto‐SCT depending on histology among PR patients at SCT. Auto‐SCT, autologous stem cell transplantation; PR, partial remission; ALK‐negative ALCL, anaplastic lymphoma kinase‐negative anaplastic large cell lymphoma; PTCL NOS, peripheral T‐cell lymphoma not otherwise specified; AITL, angioimmunoblastic T‐cell lymphoma.
**Supplemental Figure S9.** Estimated 5‐year outcomes of up‐front auto‐SCT depending on histology at SCT. Auto‐SCT, autologous stem cell transplantation; ALK‐negative ALCL, anaplastic lymphoma kinase‐negative anaplastic large cell lymphoma; PTCL NOS, peripheral T‐cell lymphoma not otherwise specified; AITL, angioimmunoblastic T‐cell lymphoma.
**Supplemental Figure S10.** Outcomes of salvage auto‐SCT depending on remission status at SCT (CR2+ vs. PR2+ vs. SD/PD). Auto‐SCT, autologous stem cell transplantation; CR, complete remission; PR, partial remission; SD, stable disease; PD, progressive disease.
**Supplemental Figure S11.** Outcomes of salvage auto‐SCT depending on metabolic remission status at SCT (CMR vs. non‐CMR). Auto‐SCT, autologous stem cell transplantation; CMR, complete metabolic remission.
**Supplemental Figure S12.** Outcomes of salvage auto‐SCT depending on CR status determined by CT and PET (CR by CT vs. CMR by PET). Auto‐SCT, autologous stem cell transplantation; CR, complete remission; CT, computed tomography; PET, positron emission tomography; CMR, complete metabolic remission.
**Supplemental Figure S13.** Outcomes of salvage auto‐SCT depending on histology among CR patients at SCT. Auto‐SCT, autologous stem cell transplantation; CR, complete remission; ALK‐negative ALCL, anaplastic lymphoma kinase‐negative anaplastic large cell lymphoma; PTCL NOS, peripheral T‐cell lymphoma not otherwise specified; AITL, angioimmunoblastic T‐cell lymphoma.
**Supplemental Figure S14.** Outcomes of salvage auto‐SCT depending on histology among CMR patients at SCT. Auto‐SCT, autologous stem cell transplantation; CMR, complete metabolic remission; ALK‐negative ALCL, anaplastic lymphoma kinase‐negative anaplastic large cell lymphoma; PTCL NOS, peripheral T‐cell lymphoma not otherwise specified; AITL, angioimmunoblastic T‐cell lymphoma.
**Supplemental Figure S15.** Outcomes of salvage auto‐SCT depending on histology among PR patients at SCT. Auto‐SCT, autologous stem cell transplantation; PR, partial remission; ALK‐negative ALCL, anaplastic lymphoma kinase‐negative anaplastic large cell lymphoma; PTCL NOS, peripheral T‐cell lymphoma not otherwise specified; AITL, angioimmunoblastic T‐cell lymphoma.
**Supplemental Figure S16.** Estimated 5‐year outcomes of salvage auto‐SCT depending on histology at SCT. Auto‐SCT, autologous stem cell transplantation; ALK‐negative ALCL, anaplastic lymphoma kinase‐negative anaplastic large cell lymphoma; PTCL NOS, peripheral T‐cell lymphoma not otherwise specified; AITL, angioimmunoblastic T‐cell lymphoma.

Supplemental Tables S12345 23December25 R2.

## Data Availability

The data that support the findings of this study are available from the corresponding author upon reasonable request.
