# Supplementary material for: Autologous stem cell transplantation in major T‐cell lymphoma entities: An analysis by the EBMT Lymphoma Working Party
Source: Hemasphere. 2026 Feb 24;10(2):e70313. doi: 10.1002/hem3.70313 (PMC12930294; doi:10.1002/hem3.70313)

Supplemental Figure S1. Estimated 5-year outcomes of all patients of the study undergoing up-front and salvage auto-SCT

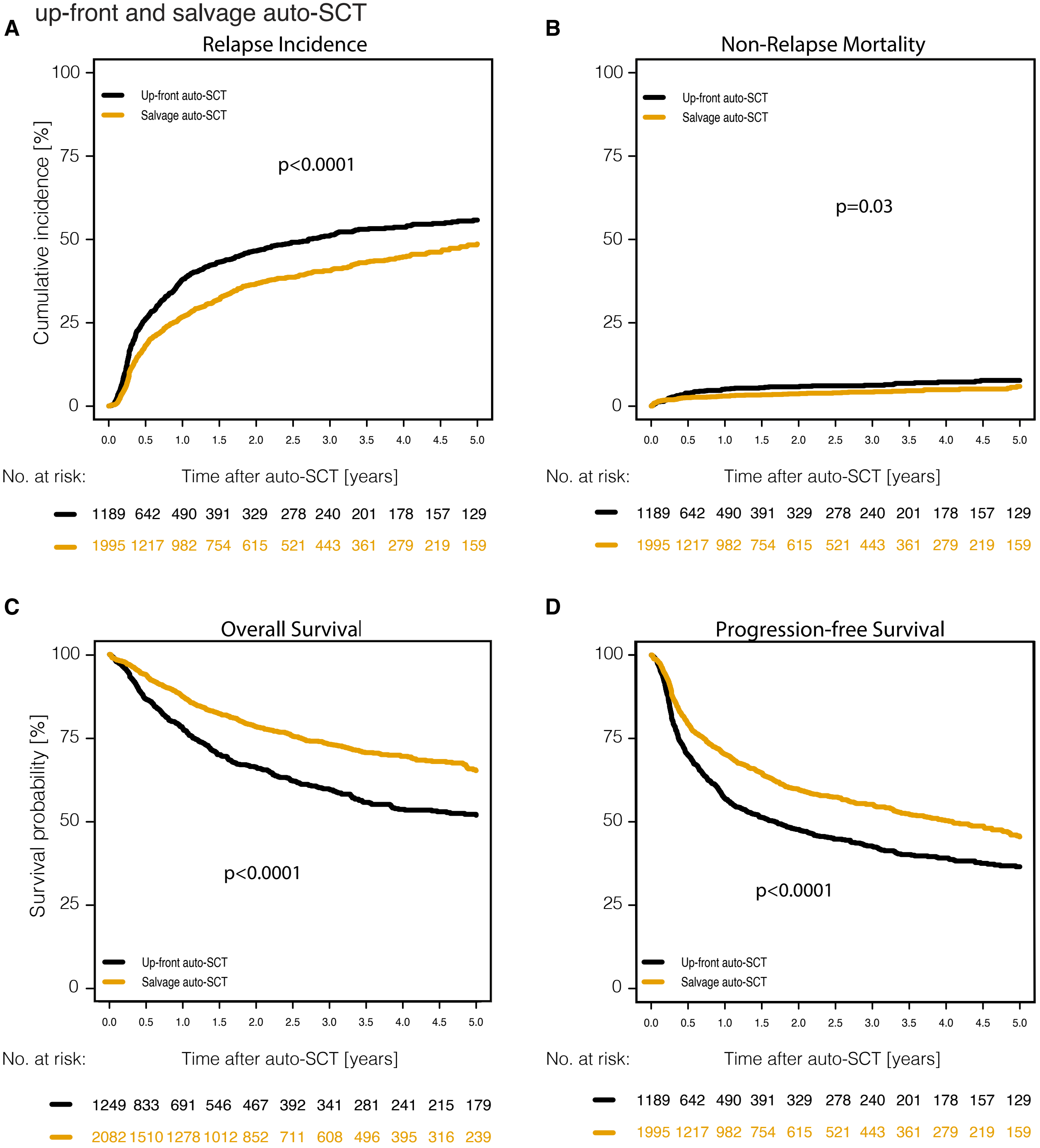

Supplemental Figure S2 (CR vs. PR at up-front auto-SCT)

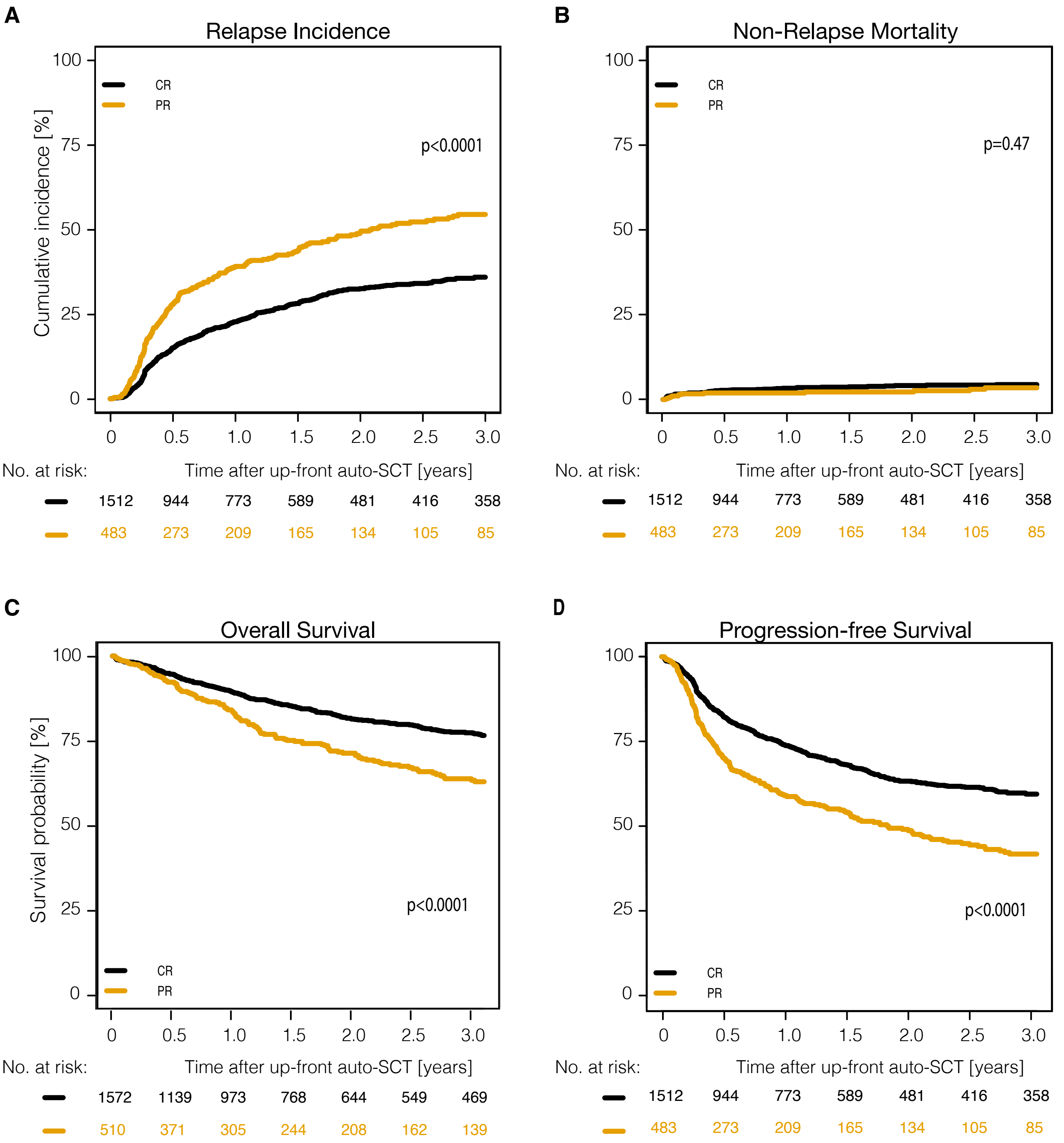

Supplemental Figure S3 (CMR vs. non-CMR at up-front auto-SCT)

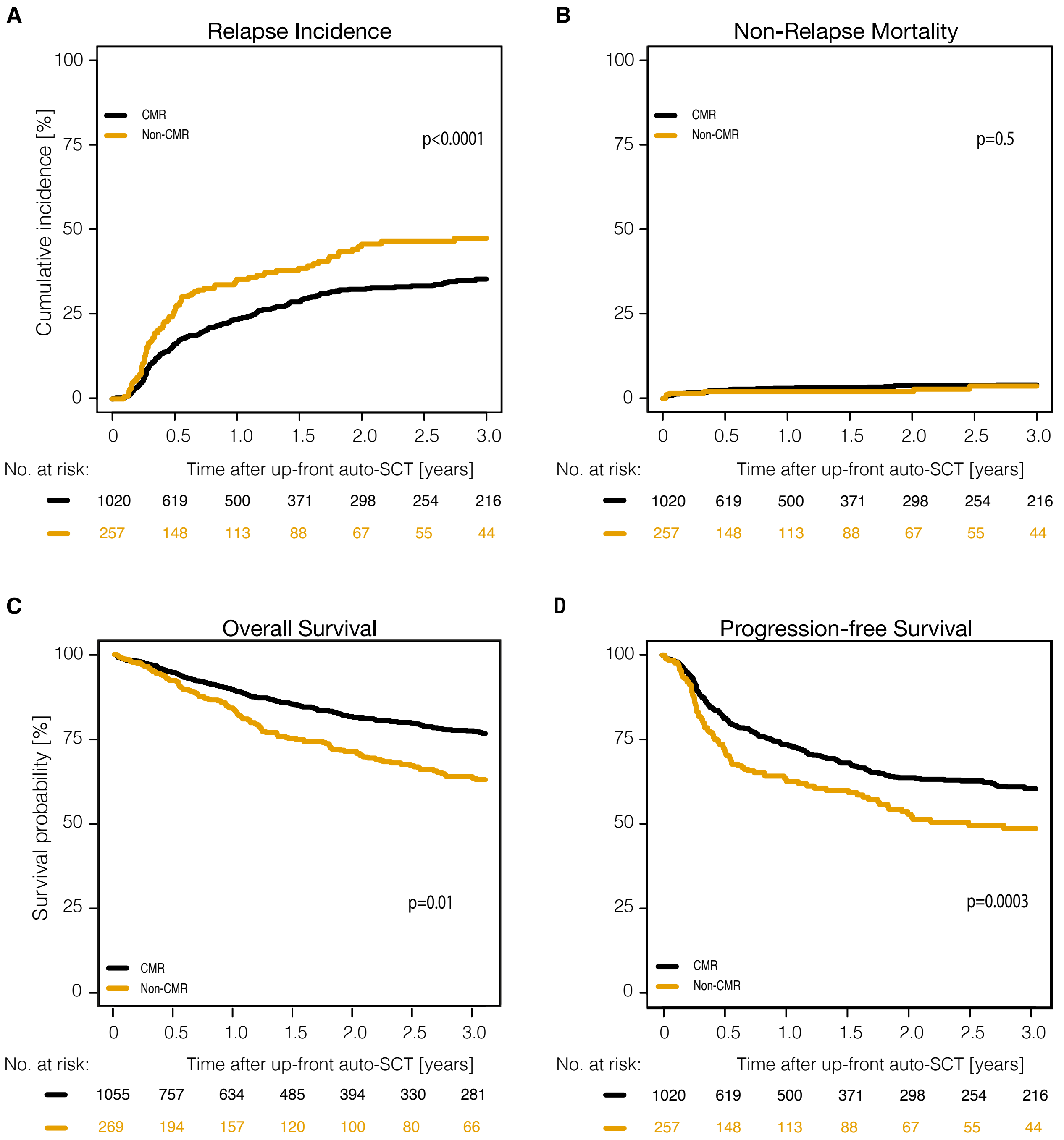

Supplemental Figure S4 (CR by CT vs. CMR by PET at up-front auto-SCT)

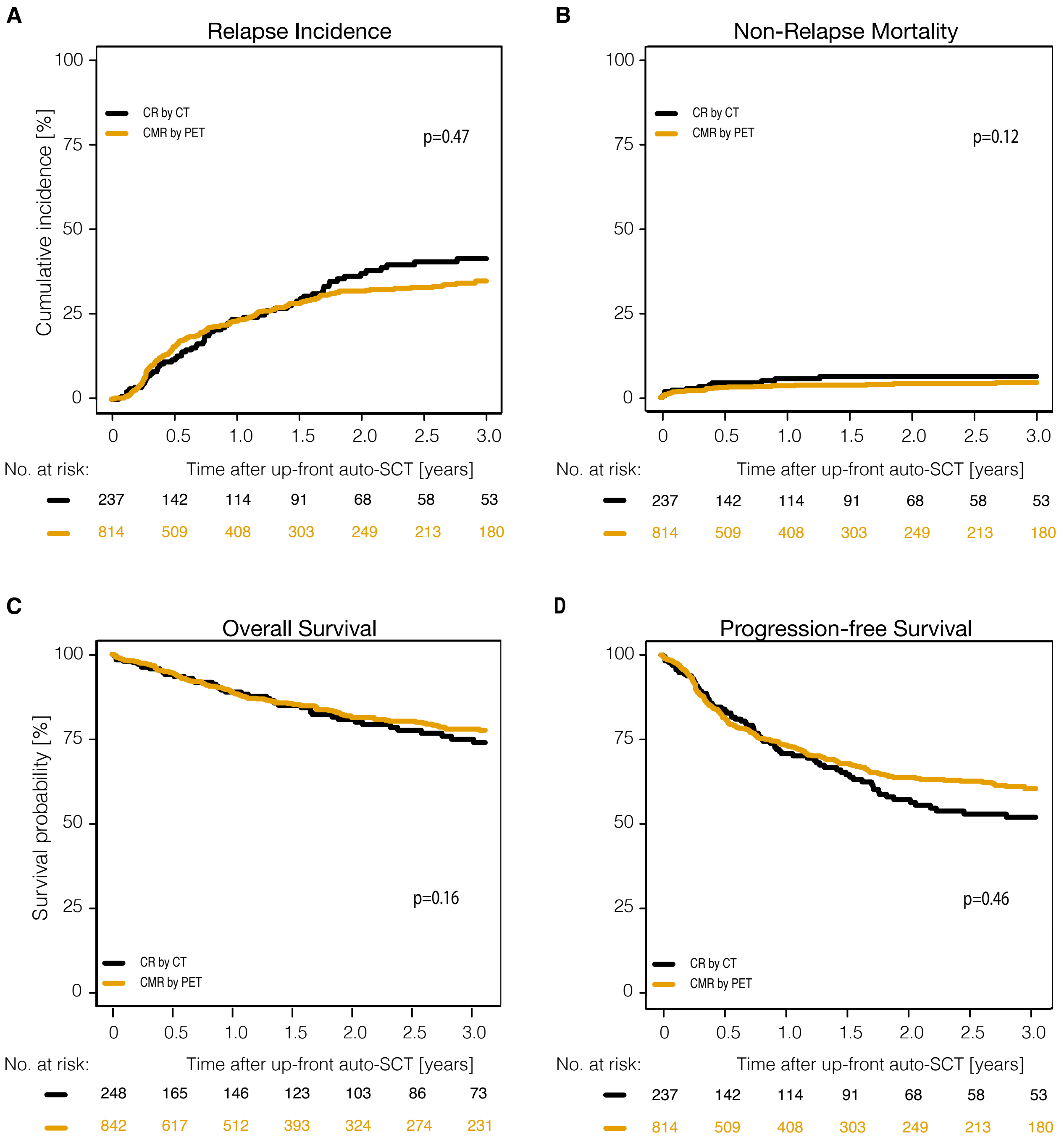

Supplemental Figure S5 (PR by CT vs. non-CMR by PET at up-front auto-SCT)

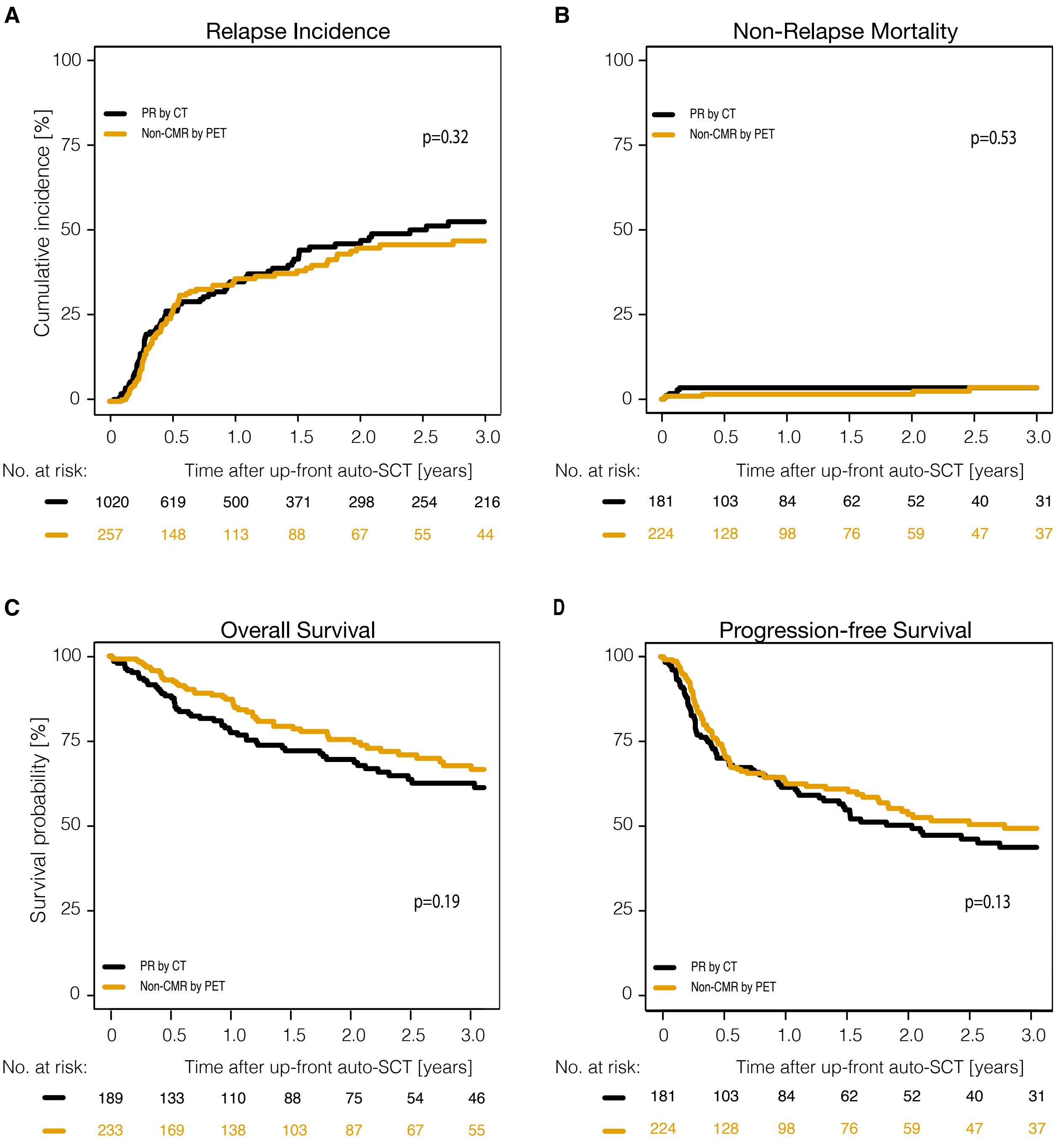

Supplemental Figure S6 (CR patients at up-front auto-SCT)

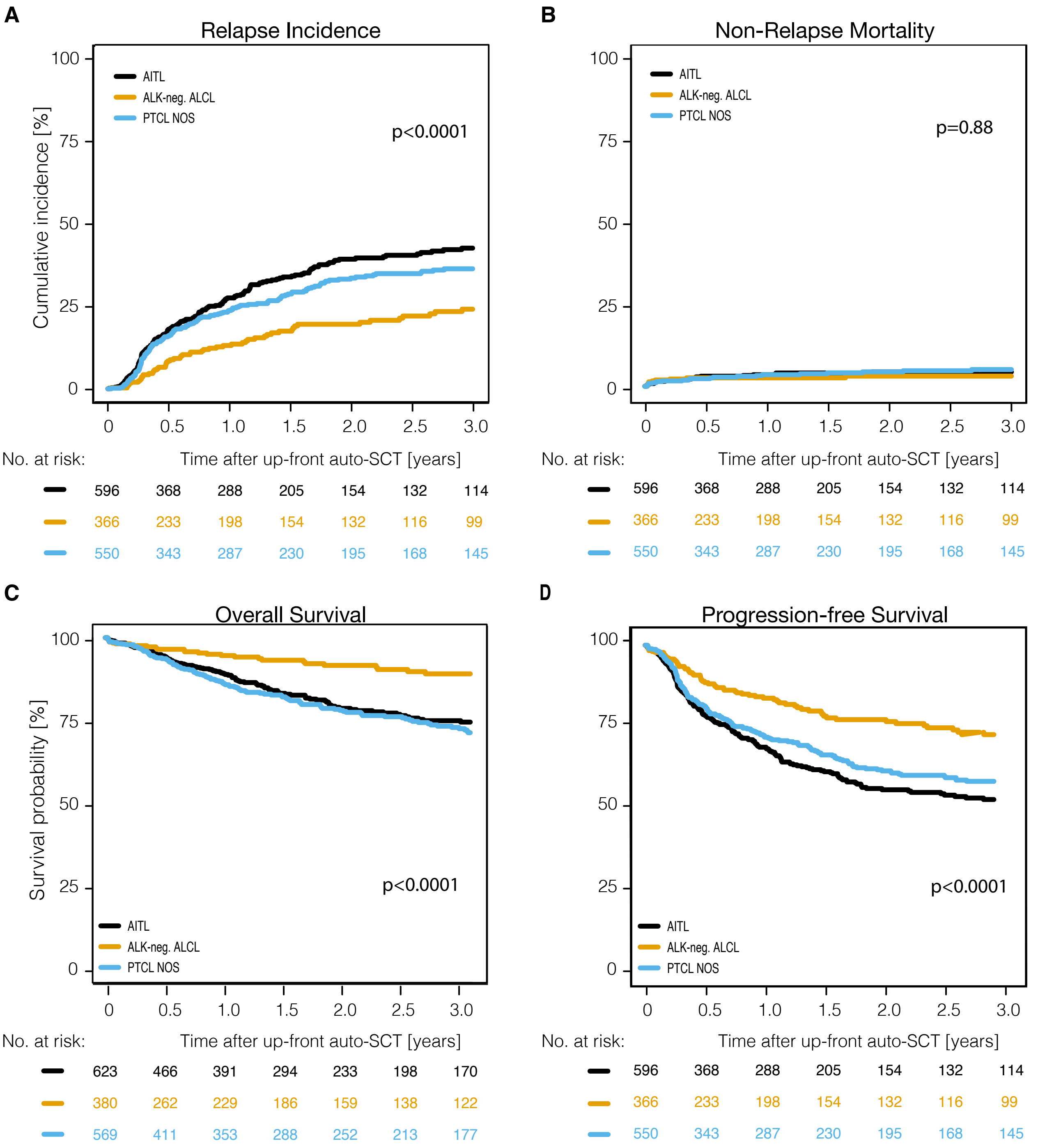

Supplemental Figure S7 (CMR patients at up-front auto-SCT)

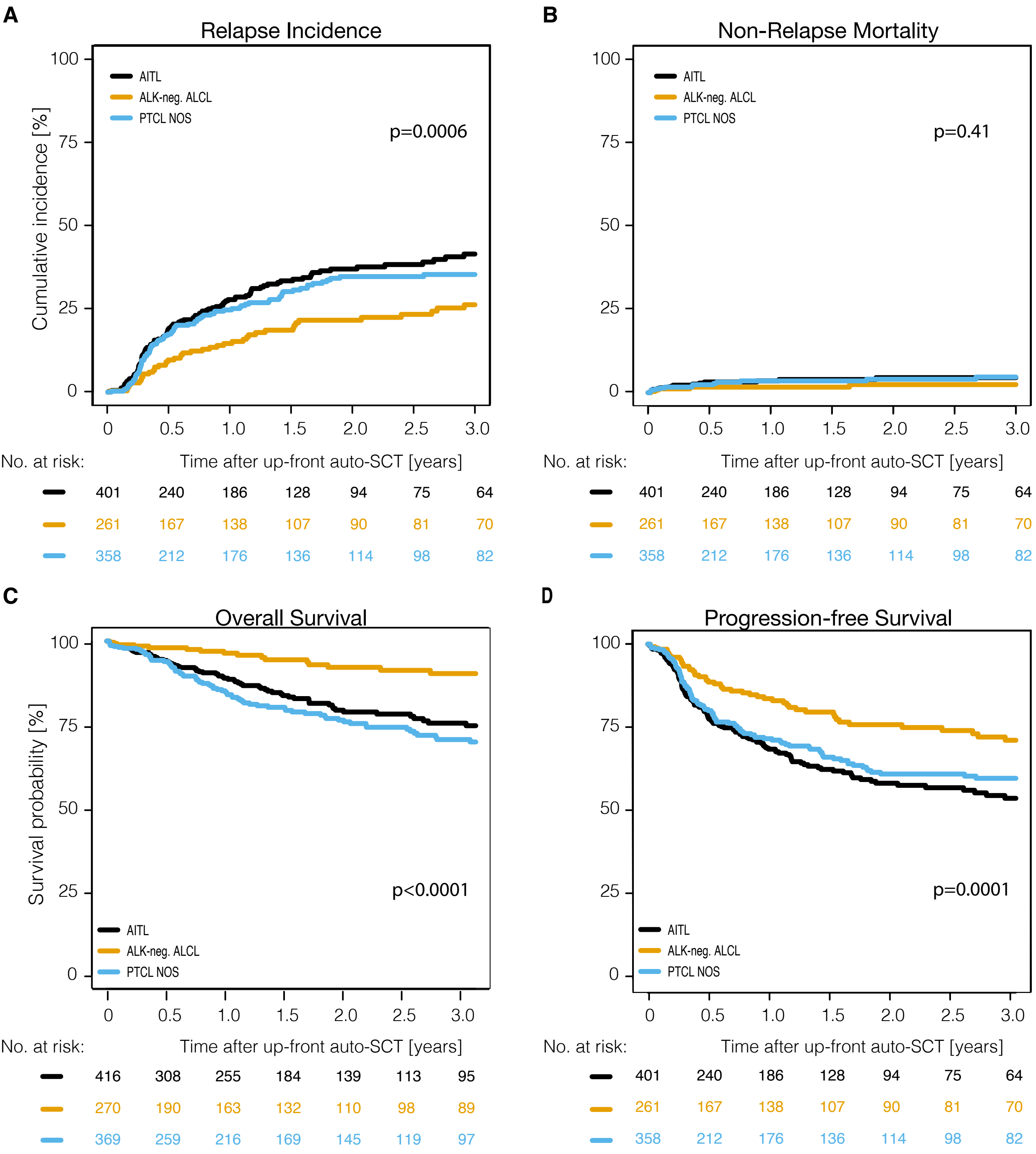

Supplemental Figure S8 (PR patients at up-front auto-SCT)

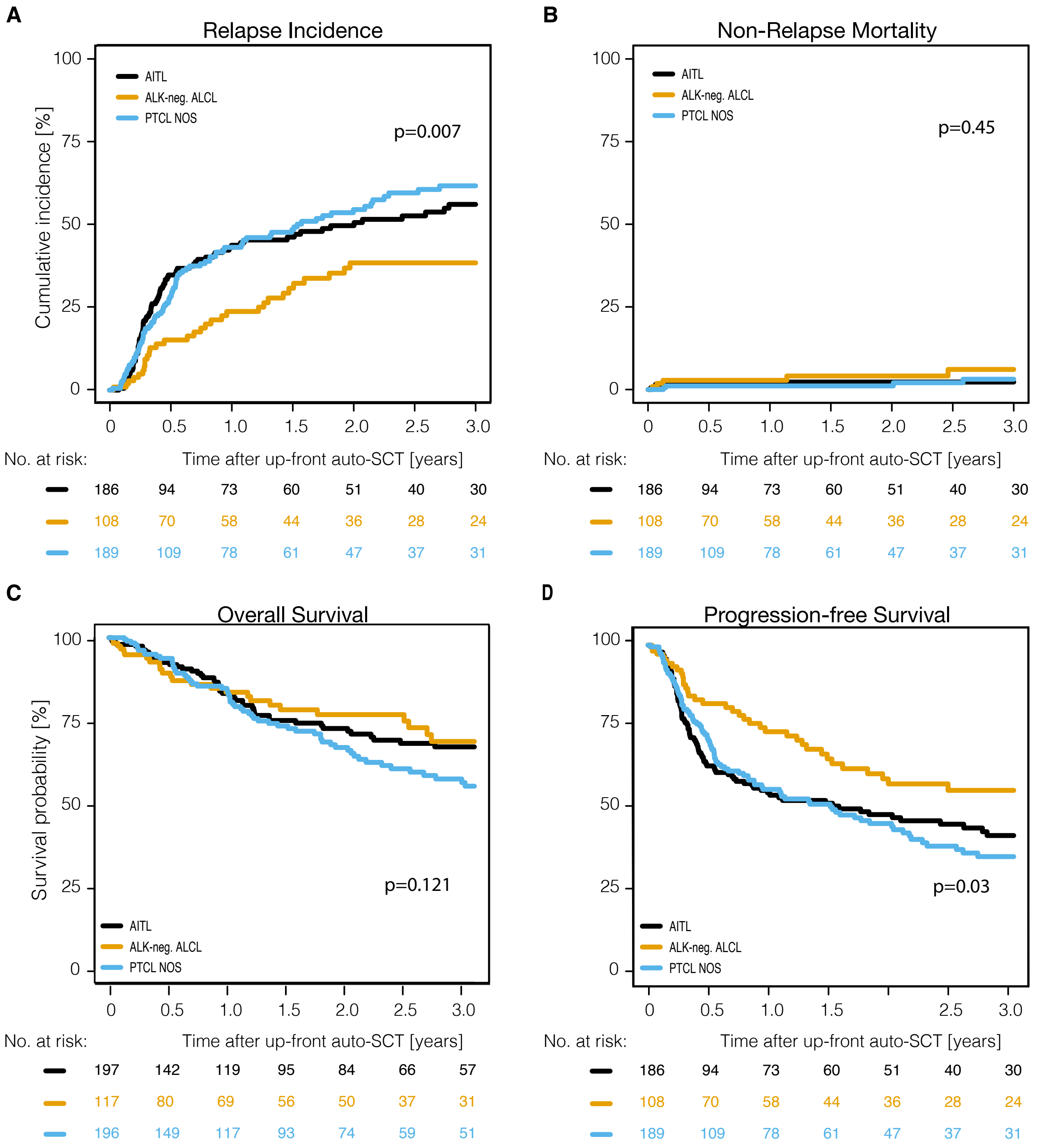

Supplemental Figure S9. Estimated 5-year outcomes of up-front auto-SCT depending on histology at SCT

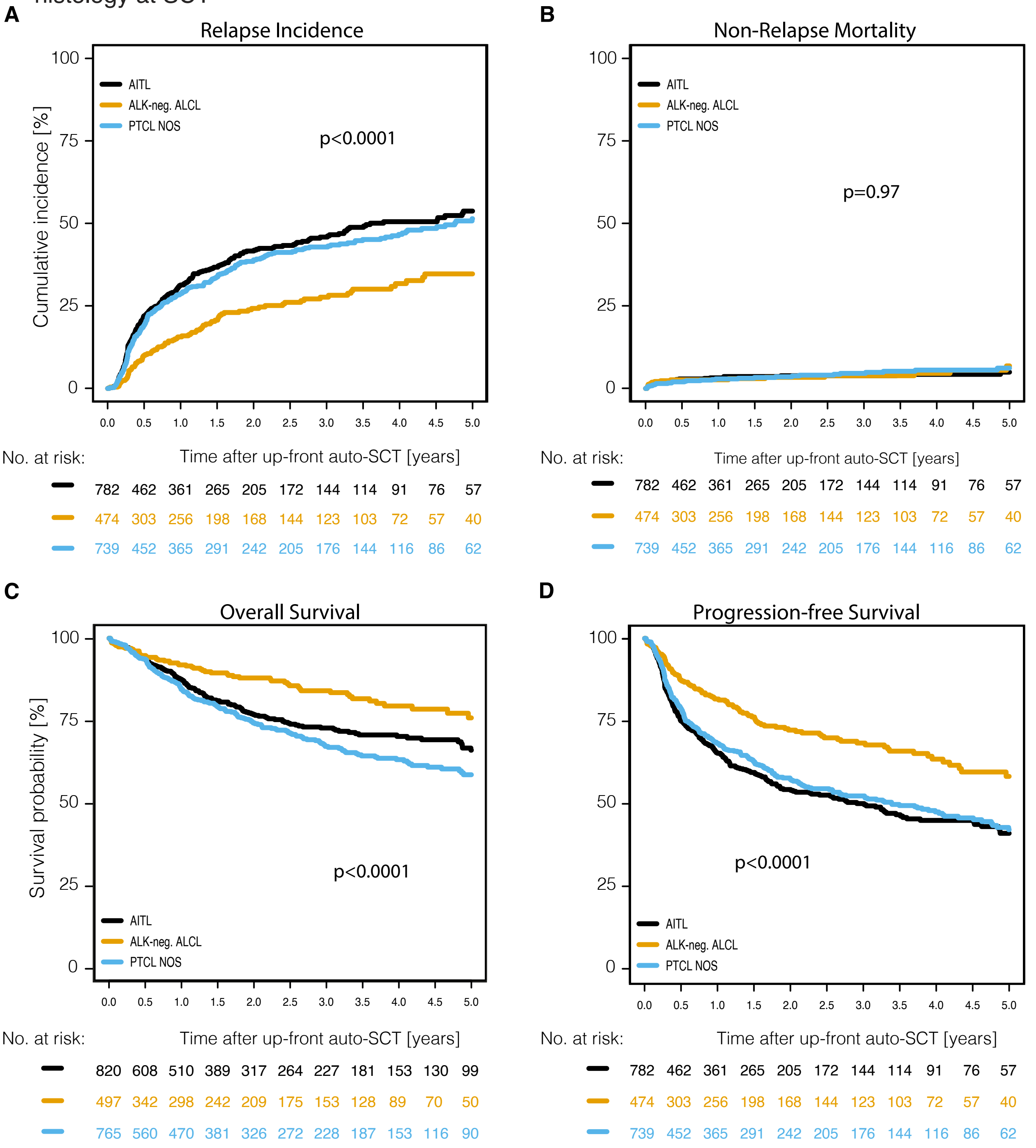

Supplemental Figure S10 (CR2+ vs. PR2+ vs. SD/PD at salvage auto-SCT)

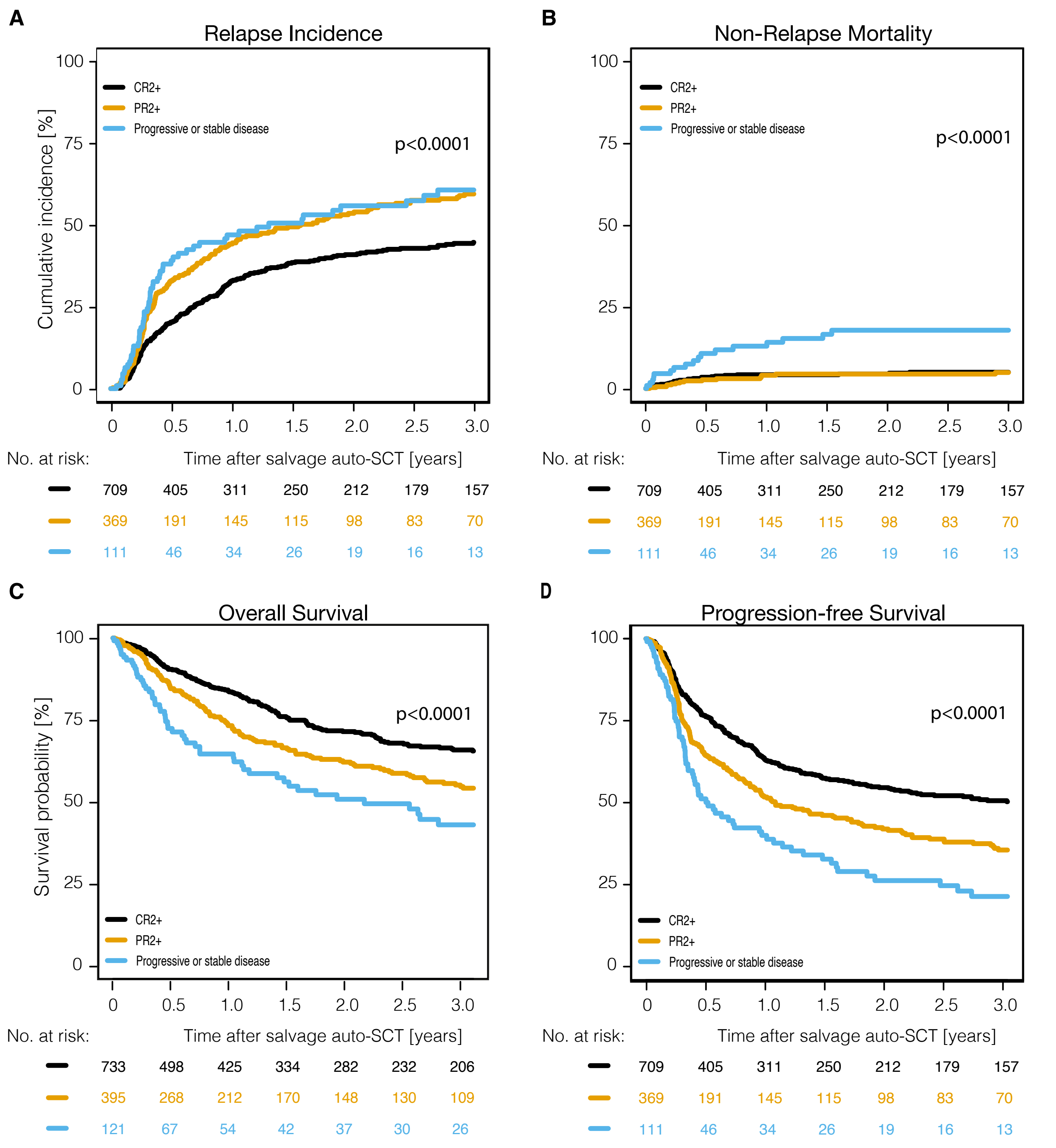

Supplemental Figure S11 (CMR vs. non-CMR at salvage auto-SCT)

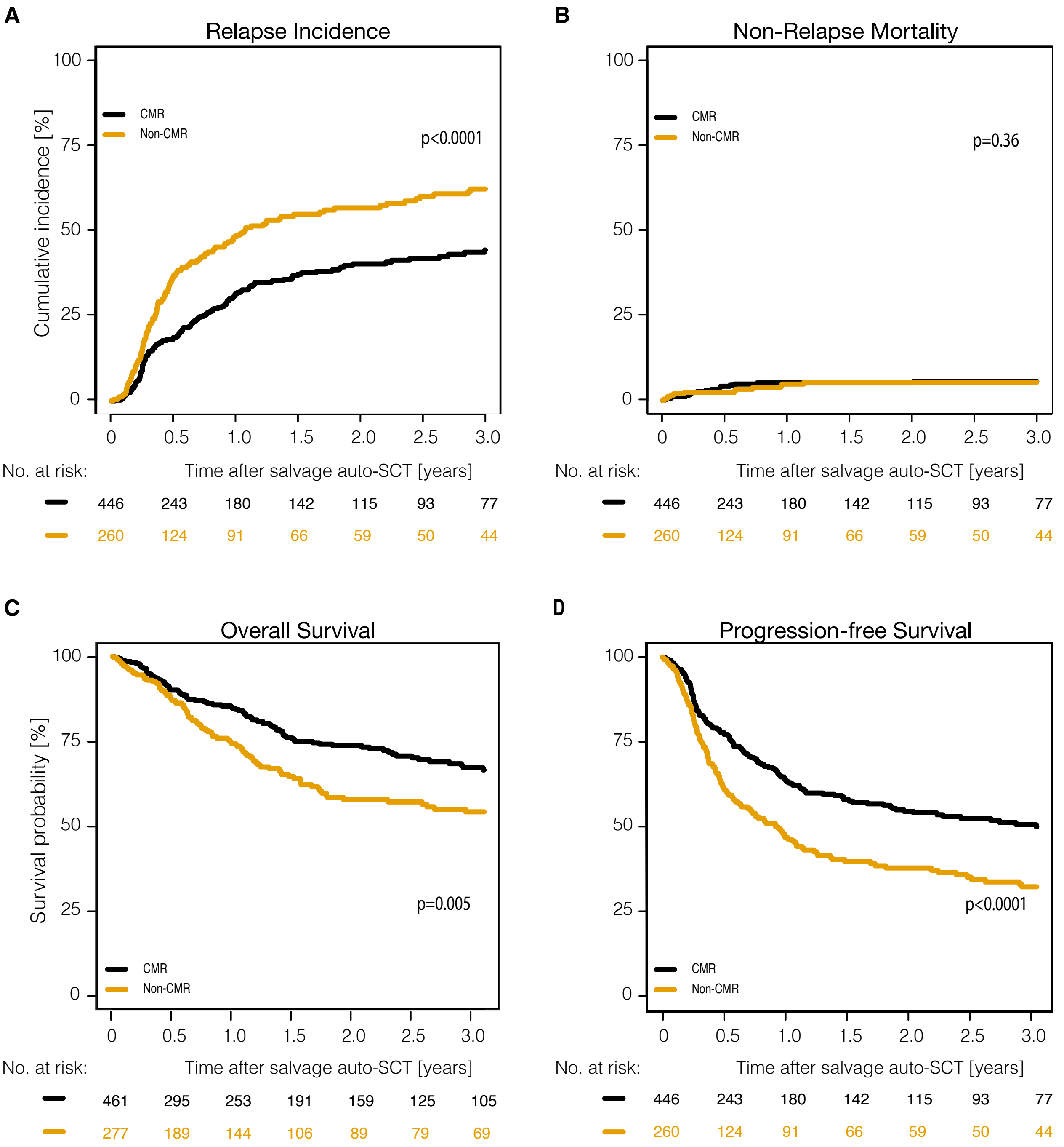

Supplemental Figure S12 (CR2+ by CT vs. CMR by PET at salvage auto-SCT)

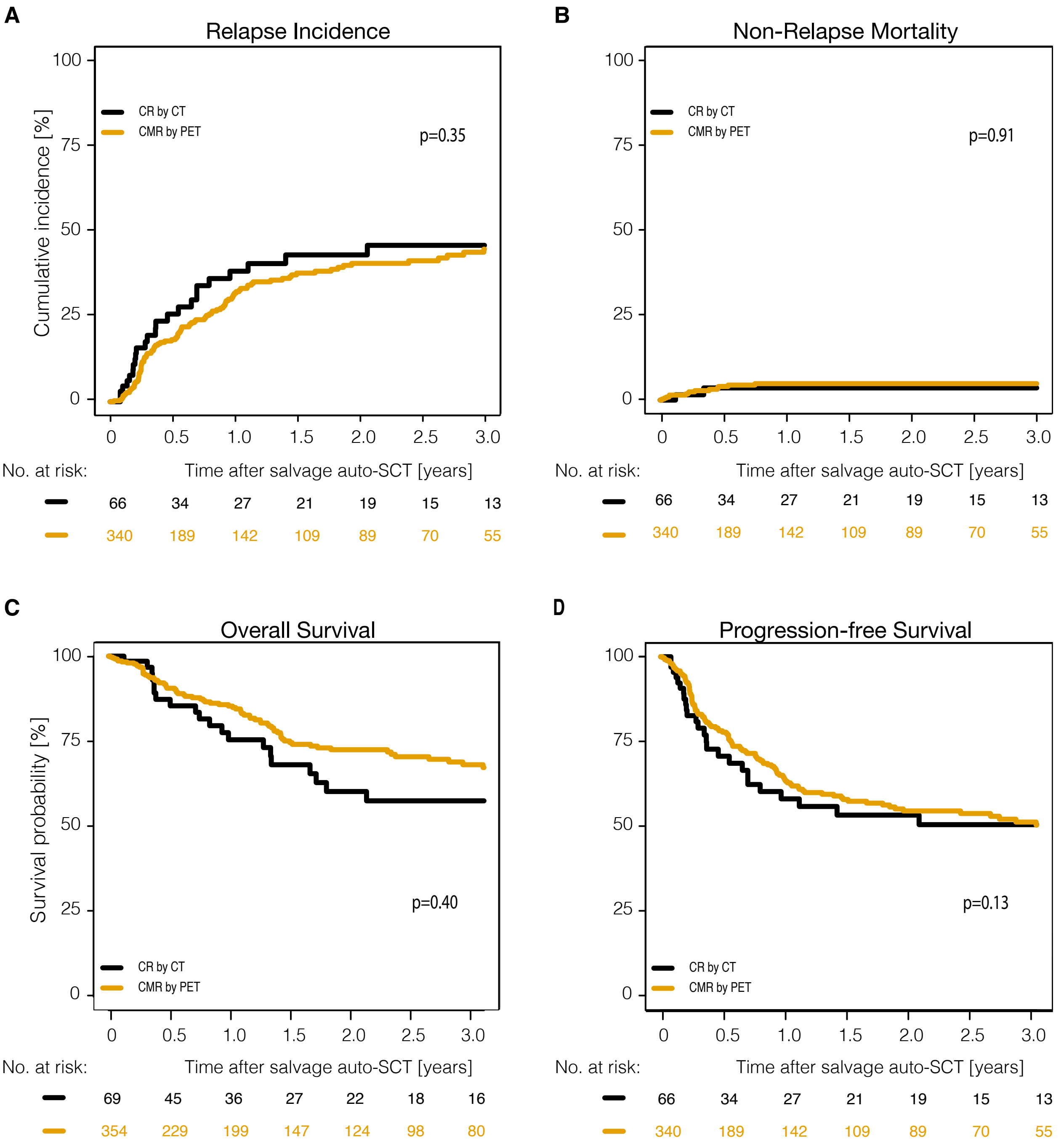

Supplemental Figure S13 (CR2+ patients at salvage auto-SCT)

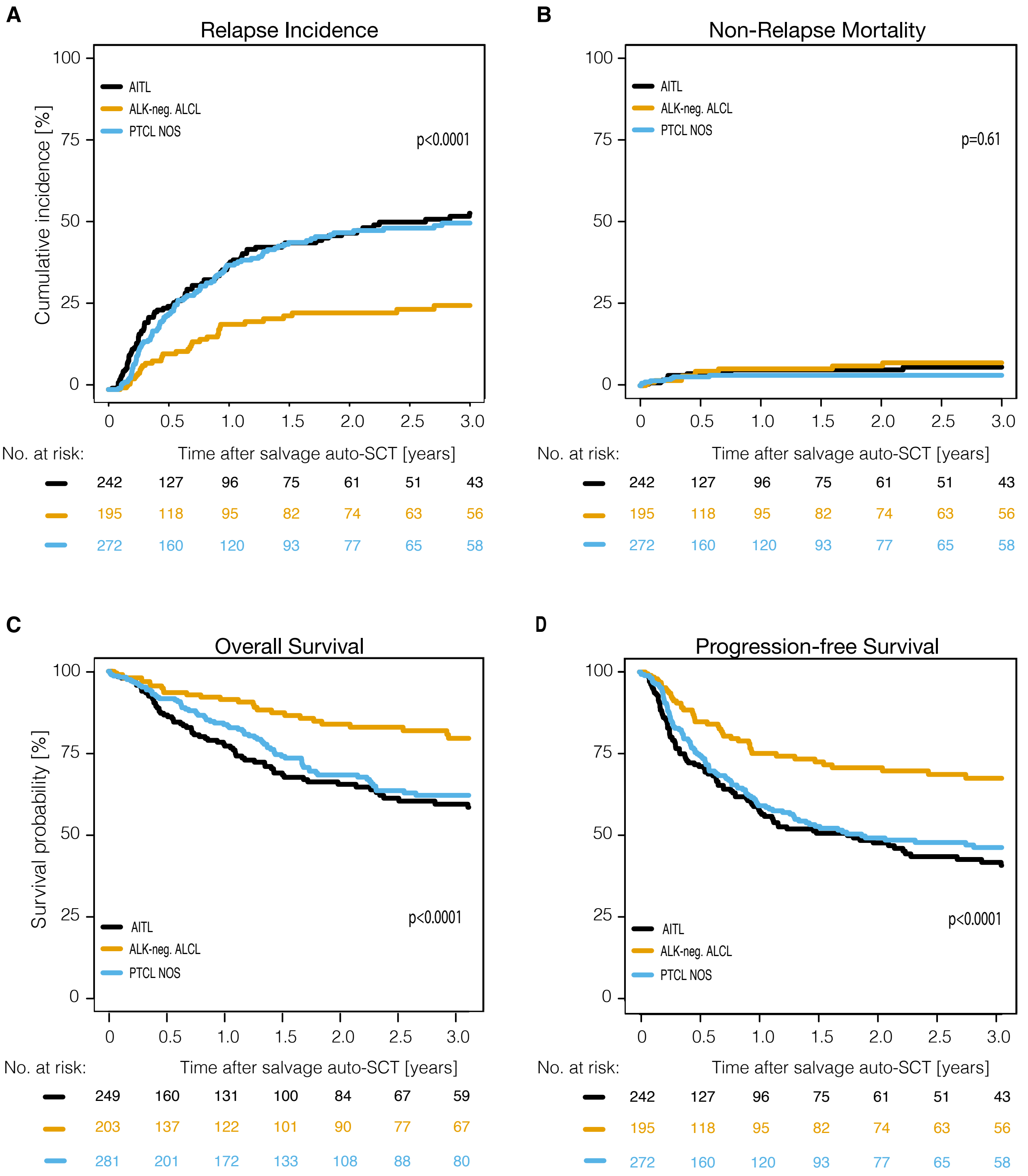

Supplemental Figure S14 (CMR patients at salvage auto-SCT)

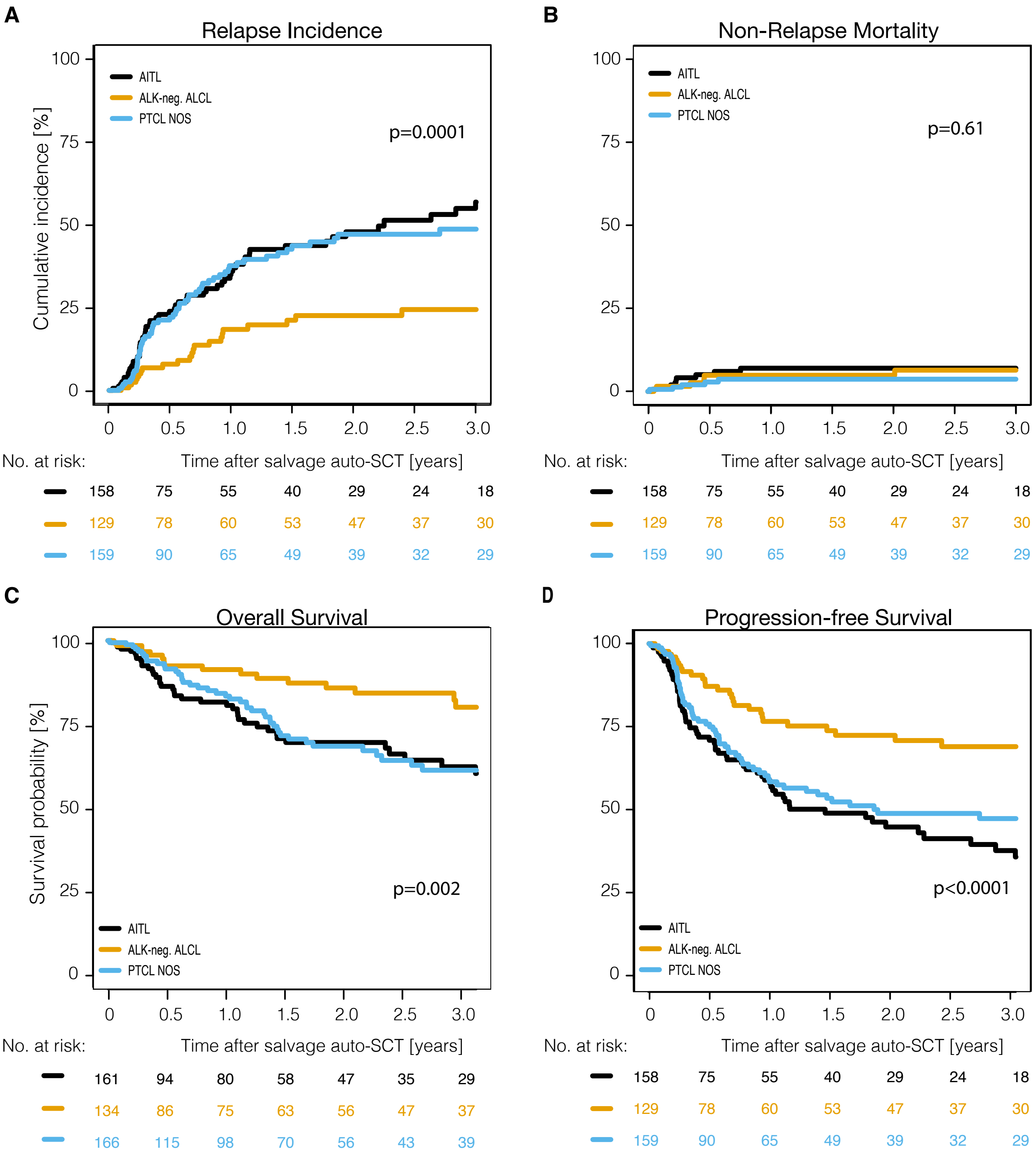

Supplemental Figure S15 (PR2+ patients at salvage auto-SCT)

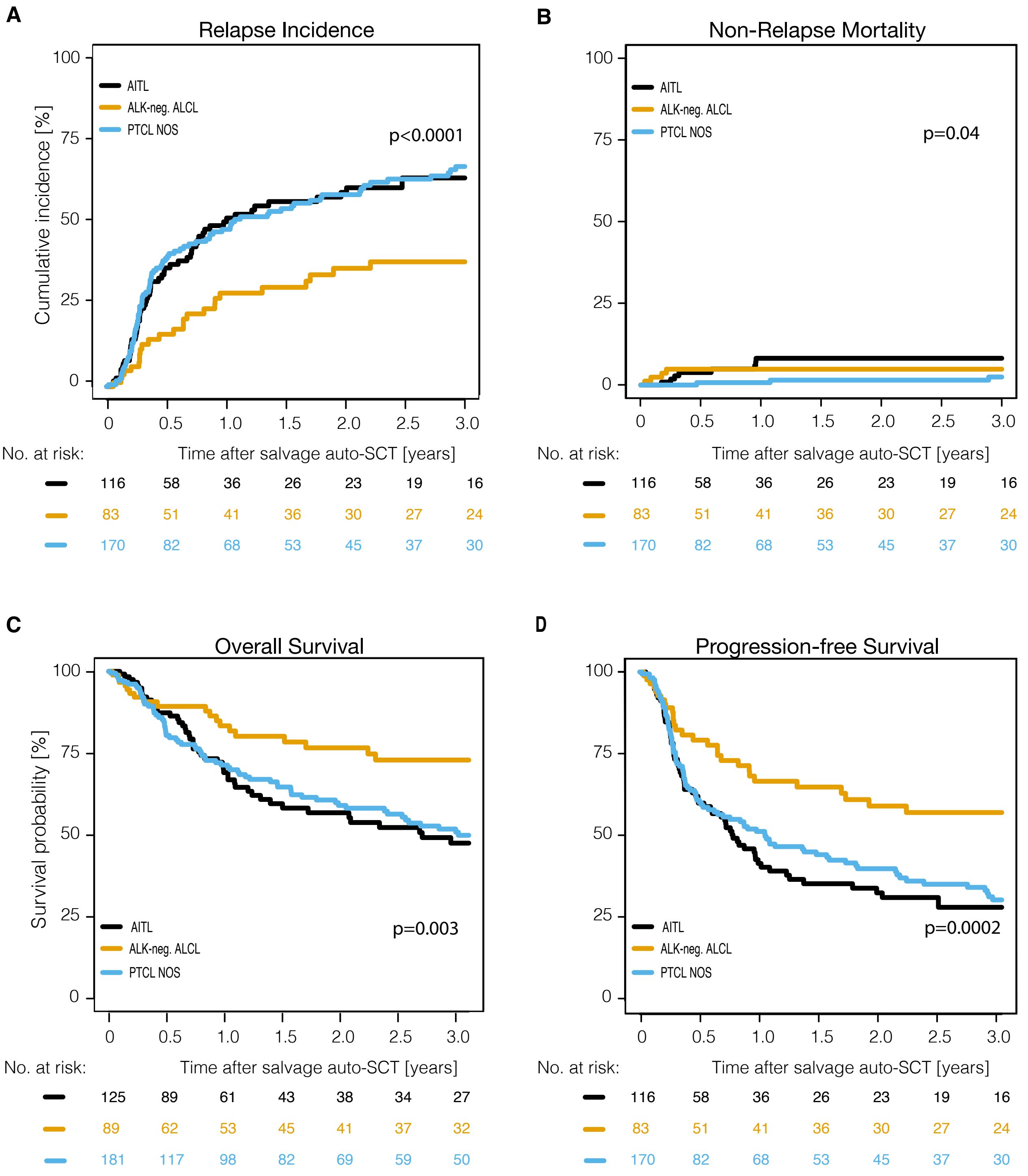

Supplemental Figure S16. Estimated 5-year outcomes of salvage auto-SCT depending on histology at SCT

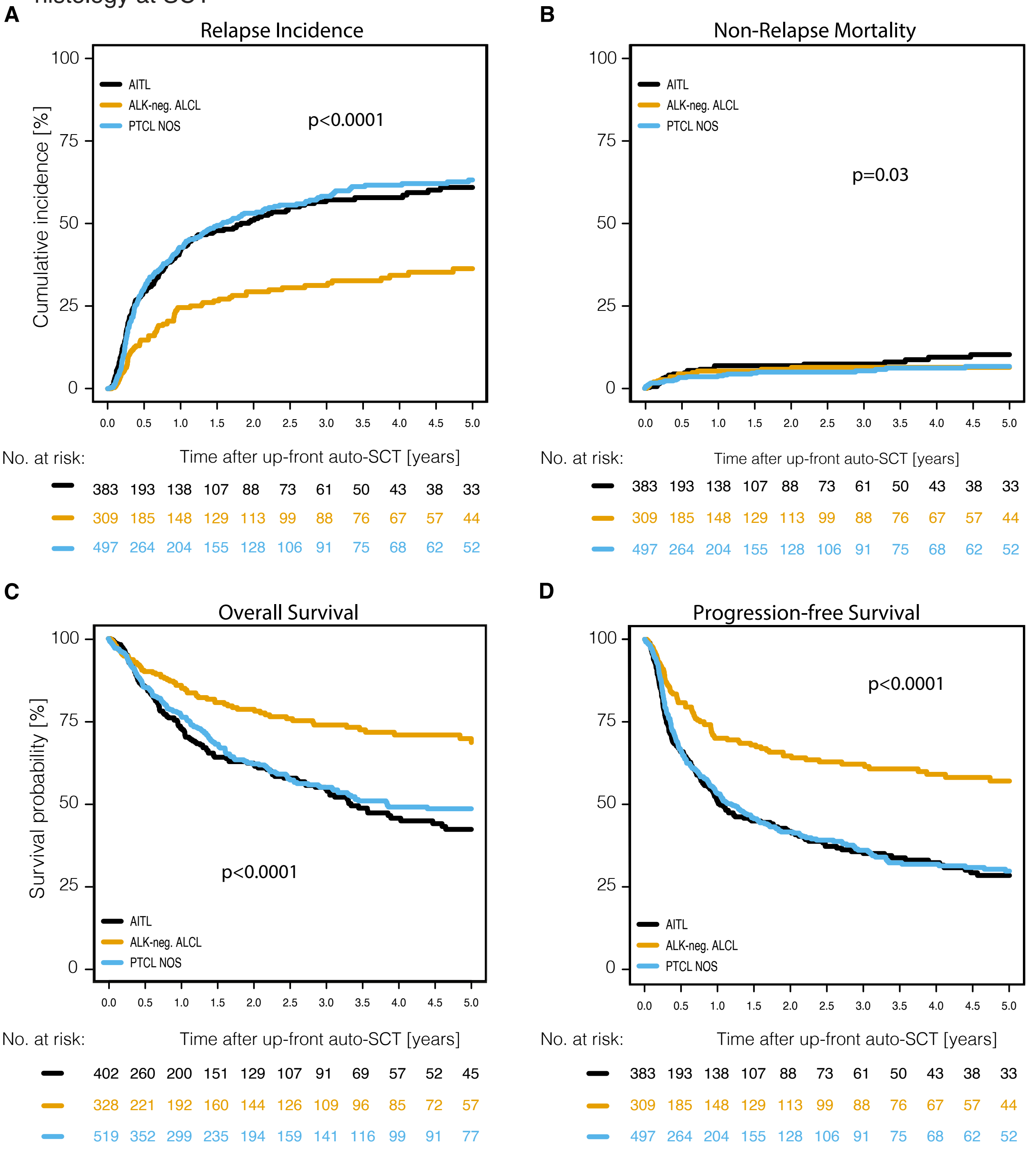

Supplement: Supplementary file 1 — Supplemental Figure S1. Estimated 5‐year outcomes of all patients of the study undergoing up‐front and salvage auto‐SCT. Auto‐SCT, autologous stem cell transplantation. Supplemental Figure S2. Outcomes of up‐front auto‐SCT depending on remission status at SCT (CR vs. PR). Auto‐SCT, autologous stem cell transplantation; CR, complete remission; PR, partial remission. Supplemental Figure S3. Outcomes of up‐front auto‐SCT depending on metabolic remission status at SCT (CMR vs. non‐CMR). Auto‐SCT, autologous stem cell transplantation; CMR, complete metabolic remission. Supplemental Figure S4. Outcomes of up‐front auto‐SCT depending on CR status determined by CT and PET (CR by CT vs. CMR by PET). Auto‐SCT, autologous stem cell transplantation; CR, complete remission; CT, computed tomography; PET, positron emission tomography; CMR, complete metabolic remission. Supplemental Figure S5. Outcomes of up‐front auto‐SCT depending on non‐CR status determined by CT and PET (PR by CT vs. non‐CMR by PET). Auto‐SCT, autologous stem cell transplantation; CR, complete remission; PR, partial remission; CT, computed tomography; CMR, complete metabolic remission; PET, positron emission tomography. Supplemental Figure S6. Outcomes of up‐front auto‐SCT depending on histology among CR patients at SCT. Auto‐SCT, autologous stem cell transplantation; CR, complete remission; ALK‐negative ALCL, anaplastic lymphoma kinase‐negative anaplastic large cell lymphoma; PTCL NOS, peripheral T‐cell lymphoma not otherwise specified; AITL, angioimmunoblastic T‐cell lymphoma. Supplemental Figure S7. Outcomes of up‐front auto‐SCT depending on histology among CMR patients at SCT. Auto‐SCT, autologous stem cell transplantation; CMR, complete metabolic remission; ALK‐negative ALCL, anaplastic lymphoma kinase‐negative anaplastic large cell lymphoma; PTCL NOS, peripheral T‐cell lymphoma not otherwise specified; AITL, angioimmunoblastic T‐cell lymphoma. Supplemental Figure S8. Outcomes of up‐front auto‐SCT dependin [file HEM3-10-e70313-s001.pdf]
