# Supplementary material for: Autologous stem cell transplantation in major T‐cell lymphoma entities: An analysis by the EBMT Lymphoma Working Party
Source: Hemasphere. 2026 Feb 24;10(2):e70313. doi: 10.1002/hem3.70313 (PMC12930294; doi:10.1002/hem3.70313)
Supplement: Supplementary file 2 — Supplemental Tables S12345 23December25 R2. [file HEM3-10-e70313-s002.docx]

## **Supplemental Table S1.** Trends of up-front and salvage auto-SCT in the investigated time period (2010-2022). Auto-SCT, autologous stem cell transplantation; CR, complete remission.

| **Variable** | **Up-front auto-SCT** | **Salvage auto-SCT** |
| --- | --- | --- |
|  | **n=2082 (100%)** | **n=1249 (100%)** |
| **Number of auto-SCT per year, n** |  |  |
| 2010 | 37 | 46 |
| 2011 | 40 | 45 |
| 2012 | 53 | 60 |
| 2013 | 41 | 45 |
| 2014 | 27 | 39 |
| 2015 | 87 | 71 |
| 2016 | 238 | 149 |
| 2017 | 278 | 170 |
| 2018 | 275 | 132 |
| 2019 | 283 | 142 |
| 2020 | 260 | 130 |
| 2021 | 261 | 140 |
| 2022 | 202 | 80 |
| **Median age at auto-SCT, range [IQR]** |  |  |
| 2010-2012 | 54.9 (18.9-73.3)  [45.6-60.1] | 54.7 (20.2-74.1)  [45.3-62.4] |
| 2013-2015 | 56.3 (20.4-70.9)  [47.5-63] | 57.9 (20.7-77.2)  [48.4-63.9] |
| 2016-2018 | 55.9 (18.3-74.1)  [47.7-62.9] | 58.4 (19.2-85.7)  [50.3-64.6] |
| 2019-2022 | 57.1 (19.2-77.3)  [49.3-63.6] | 59 (18.7-82.3)  [49.2-64.9] |
| **Proportion of patients aged ≥ 65 receiving auto-SCT, n (%)** |  |  |
| 2010-2012 | 17 (4.7%) | 22 (7.7%) |
| 2013-2015 | 25 (6.9%) | 30 (10.6%) |
| 2016-2018 | 129 (35.4%) | 111 (39.1%) |
| 2019-2022 | 193 (53%) | 121 (42.6%) |
| **Proportion of up-front and salvage auto-SCT, n (%)** |  |  |
| 2010-2012 | 130 (46.3%) | 151 (53.7%) |
| 2013-2015 | 155 (50%) | 155 (50%) |
| 2016-2018 | 791 (63.7%) | 451 (36.3%) |
| 2019-2022 | 1006 (67.2%) | 492 (32.8%) |
| **CR rate at auto-SCT, n (%)** |  |  |
| 2010-2012 | 92 (70.8%) | 84 (55.6%) |
| 2013-2015 | 109 (70.3%) | 79 (59.7%) |
| 2016-2018 | 585 (74%) | 266 (59.0%) |
| 2019-2022 | 786 (78.1%) | 304 (61.8%) |

## **Supplemental Table S2.** Outcomes of up-front auto-SCT for all patients of the study as well as separately for AITL, PTCL NOS and ALK-neg. ALCL. Auto-SCT, autologous stem cell transplantation; AITL, angioimmunoblastic T-cell lymphoma; PTCL NOS, peripheral T-cell lymphoma not otherwise specified; ALK-neg. ALCL, anaplastic lymphoma kinase-negative anaplastic large cell lymphoma.

|  | **All patients** | | **AITL** | | **PTCL NOS** | | **ALK-neg. ALCL** | |
| --- | --- | --- | --- | --- | --- | --- | --- | --- |
| **Outcomes** | **N** | **Probability [95% CI]** | **N** | **Probability [95% CI]** | **N** | **Probability [95% CI]** | **N** | **Probability [95% CI]** |
|  | **2082** |  | **820** |  | **765** |  | **497** |  |
| **Median follow-up [years]** |  | 2.1 [2.0 - 2.3] |  | 2 [1.9 - 2.2] |  | 2.6 [2.1 - 2.9] |  | 1.9 [1.5 - 2.2] |
| **Overall survival** | 2082 |  | 820 |  | 765 |  | 474 |  |
| 1 year |  | 87.4 [85.7 - 88.9] |  | 87.5 [84.8 - 89.8] |  | 84.5 [81.4 - 87.1] |  | 91.9 [88.7 - 94.2] |
| 3 years |  | 73.1 [70.5 - 75.4] |  | 72.7 [68.6 - 76.4] |  | 67.2 [62.8 - 71.2] |  | 84.1 [79.3 - 87.8] |
| **Progression-free survival** | 1995 |  | 782 |  | 739 |  | 474 |  |
| 1 year |  | 70.2 [68.0 - 72.4] |  | 65.4 [61.6 - 68.9] |  | 68.4 [64.5 - 71.9] |  | 81.8 [77.4 - 85.3] |
| 3 years |  | 55.2 [52.5 - 57.9] |  | 50.3 [45.8 - 54.5] |  | 52.6 [48.1 - 56.8] |  | 68.5 [62.8 - 73.5] |
| **Relapse incidence** | 1995 |  | 782 |  | 739 |  | 474 |  |
| 1 year |  | 26.8 [24.7 - 29] |  | 31.4 [27.8 - 35] |  | 28.7 [25.2 - 32.4] |  | 15.6 [12.1 - 19.6] |
| 3 years |  | 40.6 [37.9 - 43.2] |  | 45.9 [41.5 - 50.1] |  | 42.9 [38.6 - 47.1] |  | 27.7 [22.6 - 32.9] |
| **Non-relapse mortality** | 1995 |  | 782 |  | 739 |  | 474 |  |
| 1 year |  | 3.0 [2.3 - 3.8] |  | 3.2 [2.1 - 4.7] |  | 2.9 [1.8 - 4.4] |  | 2.6 [1.4 - 4.4] |
| 3 years |  | 4.2 [3.2 - 5.3] |  | 3.9 [2.6 - 5.6] |  | 4.6 [3 - 6.6] |  | 3.9 [2.2 - 6.3] |

## **Supplemental Table S3.** Multivariate Cox proportional hazards models of outcomes of up-front auto-SCT for all patients (ALK-neg. ALCL, PTCL NOS, AITL). Auto-SCT, autologous stem cell transplantation; HR=hazard ratio; CI=confidence interval; ALK-neg. ALCL, anaplastic lymphoma kinase-negative anaplastic large cell lymphoma; AITL, angioimmunoblastic T-cell lymphoma; PTCL NOS, peripheral T-cell lymphoma not otherwise specified; PR, partial remission; CR, complete remission; BEAM, carmustine, etoposide, cytarabine, melphalan. References for Cox proportional hazards are highlighted in **bold**.

|  | **Overall Survival** | | **Progression-free Survival** | | **Relapse Incidence** | | **Non-relapse Mortality** | |
| --- | --- | --- | --- | --- | --- | --- | --- | --- |
| **Variable** | **HR [95% CI]** | **P-value** | **HR [95% CI]** | **P-value** | **HR [95% CI]** | **P-value** | **HR [95% CI]** | **P-value** |
| Year of SCT (by 2 years) | 1.08 (0.99-1.17) | 0.08 | 1.04 (0.98-1.11) | 0.15 | 1.05 (0.98-1.12) | 0.14 | 1.01 (0.85-1.21) | 0.9 |
| Sex: female vs. **male** | 0.73 (0.59-0.9) | **0.003** | 0.8 (0.68-0.94) | **0.006** | 0.79 (0.66-0.93) | **0.006** | 0.89 (0.55-1.42) | 0.61 |
| Karnofsky index ≥ 90% vs. **<90%** | 0.83 (0.65-1.05) | 0.13 | 1.03 (0.86-1.25) | 0.73 | 1.1 (0.89-1.35) | 0.37 | 0.73 (0.44-1.21) | 0.22 |
| Lymphoma: ALK-neg. ALCL vs. **AITL** | 0.69 (0.51-0.95) | **0.02** | 0.56 (0.44-0.7) | **<0.001** | 0.5 (0.39-0.64) | **<0.001** | 1.08 (0.58-2.01) | 0.81 |
| Lymphoma: PTCL NOS vs. **AITL** | 1.3 (1.04-1.61) | **0.02** | 0.93 (0.78-1.1) | 0.38 | 0.9 (0.75-1.08) | 0.25 | 1.17 (0.69-1.96) | 0.56 |
| Age at auto-SCT (by ten years) | 1.41 (1.27-1.57) | **<0.001** | 1.14 (1.06-1.22) | **<0.001** | 1.07 (0.99-1.16) | 0.07 | 2.08 (1.57-2.75) | **<0.001** |
| Status at auto-SCT: PR vs. **CR** | 1.75 (1.42-2.17) | **<0.001** | 1.74 (1.48-2.05) | **<0.001** | 1.89 (1.59-2.24) | **<0.001** | 0.88 (0.51-1.54) | 0.66 |
| Conditioning: non-BEAM vs. **BEAM** | 1.05 (0.83-1.33) | 0.69 | 0.97 (0.82-1.15) | 0.71 | 1.01 (0.84-1.21) | 0.95 | 0.67 (0.39-1.17) | 0.16 |
| Global test χ² |  | 9.610 |  | 31.044 |  | 62.637 |  | 0.351 |
| Global p value |  | **0.03** |  | **<0.001** |  | **<0.001** |  | 0.84 |

## **Supplemental Table S4.** Outcomes of salvage auto-SCT for all patients and separately for AITL, PTCL NOS and ALK-neg. ALCL. Auto-SCT, autologous stem cell transplantation; AITL, angioimmunoblastic T-cell lymphoma; PTCL NOS, peripheral T-cell lymphoma not otherwise specified; ALK-neg. ALCL, anaplastic lymphoma kinase-negative anaplastic large cell lymphoma.

|  | **All patients** | | **AITL** | | **PTCL NOS** | | **ALK-neg. ALCL** | |
| --- | --- | --- | --- | --- | --- | --- | --- | --- |
| **Outcomes** | **N** | **Probability [95% CI]** | **N** | **Probability [95% CI]** | **N** | **Probability [95% CI]** | **N** | **Probability [95% CI]** |
|  | **1128** |  | **374** |  | **462** |  | **292** |  |
| **Median follow-up [years]** |  | 2.6 (2.2 - 3.0) |  | 2 (1.7 - 2.7) |  | 3 (2.3 - 3.5) |  | 2.5 (2 - 3.1) |
| **Overall survival** | 1128 |  | 374 |  | 462 |  | 292 |  |
| 1 year |  | 77.9 (75.2 - 80.3) |  | 72.7 (67.4 - 77.3) |  | 76.9 (72.6 - 80.6) |  | 85.8 (81.1 - 89.5) |
| 3 years |  | 59.5 (56.1 - 62.8) |  | 54.3 (47.9 - 60.3) |  | 54.9 (49.5 - 59.9) |  | 73.8 (67.4 - 79.1) |
| **Progression-free survival** | 1078 |  | 358 |  | 442 |  | 278 |  |
| 1 year |  | 57.1 (53.9 - 60.1) |  | 51.2 (45.4 - 56.6) |  | 53.7 (48.8 - 58.3) |  | 70.2 (64 - 75.5) |
| 3 years |  | 42.6 (39.2 - 46.0) |  | 35.5 (29.6 - 41.3) |  | 36.4 (31.5 - 41.4) |  | 62.4 (55.6 - 68.4) |
| **Relapse incidence** | 1078 |  | 358 |  | 442 |  | 278 |  |
| 1 year |  | 37.9 (34.9 - 41.0) |  | 42 (36.4 - 47.4) |  | 42.8 (38 - 47.5) |  | 24.6 (19.4 - 30.1) |
| 3 years |  | 51.2 (47.7 – 54.5) |  | 57.2 (50.9 - 63) |  | 58.3 (53 - 63.2) |  | 31.3 (25.2 - 37.4) |
| **Non-relapse mortality** | 1078 |  | 358 |  | 442 |  | 278 |  |
| 1 year |  | 5.0 (3.8 – 6.5) |  | 6.9 (4.4 - 10) |  | 3.5 (2.1 - 5.5) |  | 5.2 (3 - 8.4) |
| 3 years |  | 6.2 (4.8 - 7.9) |  | 7.4 (4.8 - 10.7) |  | 5.3 (3.4 - 7.8) |  | 6.3 (3.7 - 9.9) |

## **Supplemental Table S5**. Multivariate Cox proportional hazards models of outcomes of salvage auto-SCT for all patients (ALK-neg. ALCL, PTCL NOS, AITL). Auto-SCT, autologous stem cell transplantation; HR=hazard ratio; CI=confidence interval; ALK-neg. ALCL, anaplastic lymphoma kinase-negative anaplastic large cell lymphoma; AITL, angioimmunoblastic T-cell lymphoma; PTCL NOS, peripheral T-cell lymphoma not otherwise specified; Mo., months; 3+, three or more therapy lines; PR2+, second partial remission or later; CR2, second complete remission or later; SD/PD, stable disease/progressive disease; BEAM, carmustine, etoposide, cytarabine, melphalan. References for Cox proportional hazards are highlighted in **bold**.

|  | **Overall Survival** | | **Progression-free Survival** | | **Relapse Incidence** | | **Non-relapse Mortality** | |
| --- | --- | --- | --- | --- | --- | --- | --- | --- |
| **Variable** | **HR [95% CI]** | **P-value** | **HR [95% CI]** | **P-value** | **HR [95% CI]** | **P-value** | **HR [95% CI]** | **P-value** |
| Year of SCT (by 2 years) | 1.05 (0.98-1.13) | 0.15 | 1.07 (1.01-1.14) | **0.02** | 1.09 (1.03-1.16) | **0.005** | 0.94 (0.8-1.12) | 0.49 |
| Sex: female vs. **male** | 0.85 (0.68-1.06) | 0.15 | 0.82 (0.68-0.99) | **0.04** | 0.78 (0.64-0.95) | **0.01** | 1.13 (0.68-1.9) | 0.64 |
| Karnofsky index ≥ 90% vs. **<90%** | 0.75 (0.61-0.92) | **0.007** | 0.83 (0.69-1.0) | 0.053 | 0.88 (0.72-1.07) | 0.20 | 0.62 (0.37-1.03) | 0.06 |
| Lymphoma: ALK-neg. ALCL vs. **AITL** | 0.51 (0.38-0.69) | **<0.001** | 0.46 (0.35-0.59) | **<0.001** | 0.43 (0.33-0.57) | **<0.001** | 0.62 (0.33-1.19) | 0.15 |
| Lymphoma: PTCL NOS vs. **AITL** | 0.89 (0.71-1.12) | 0.31 | 0.91 (0.75-1.11) | 0.35 | 0.96 (0.78-1.18) | 0.69 | 0.63 (0.35-1.12) | 0.12 |
| Age at auto-SCT (by ten years) | 1.26 (1.15-1.38) | **<0.001** | 1.07 (0.99-1.16) | 0.08 | 1.07 (0.98-1.16) | 0.11 | 1.09 (0.88-1.36) | 0.43 |
| Time from first diagnosis to salvage auto-SCT: >12 vs. **≤12 months** | 0.97 (0.79-1.19) | 0.78 | 1.02 (0.85-1.22) | 0.82 | 1.04 (0.86-1.25) | 0.67 | 0.98 (0.59-1.63) | 0.93 |
| Number of therapy lines at auto-SCT: 3+ vs. **2** | 0.96 (0.77-1.2) | 0.72 | 1.14 (0.95-1.37) | 0.16 | 1.12 (0.92-1.36) | 0.25 | 1.19 (0.71-2.01) | 0.51 |
| Status at auto-SCT: PR2+ vs. **CR2+** | 1.44 (1.16-1.79) | **<0.001** | 1.42 (1.18-1.72) | **<0.001** | 1.45 (1.19-1.77) | **<0.001** | 1.11 (0.61-2.04) | 0.73 |
| Status at auto-SCT: SD/PD+ vs. **CR2+** | 2.08 (1.52-2.85) | **<0.001** | 2.1 (1.6-2.76) | **<0.001** | 1.81 (1.33-2.45) | **<0.001** | 4.38 (2.34-8.2) | **<0.001** |
| Conditioning: non-BEAM vs. **BEAM** | 1.1 (0.89-1.35) | 0.38 | 1.02 (0.85-1.23) | 0.82 | 0.93 (0.77-1.13) | 0.49 | 1.59 (0.96-2.64) | 0.07 |
| Global test χ² |  | 23.272 |  | 75.233 |  | 1.771 |  | 12.652 |
| Global p value |  | **<0.001** |  | **<0.001** |  | 1.0 |  | 0.076 |
